# Functional characterization and comparison of lycopene epsilon-cyclase genes in *Nicotiana tabacum*

**DOI:** 10.1186/s12870-022-03634-5

**Published:** 2022-05-21

**Authors:** Weina Song, Fang Wei, Shuwen Gao, Chen Dong, Jianfeng Hao, Lifeng Jin, Feng Li, Pan Wei, Jinggong Guo, Ran Wang

**Affiliations:** 1 College of Life Sciences, Henan Agricultural University, Zhengzhou, 450002 Henan China; 2 School of Life Sciences, Zhengzhou University, Zhengzhou, 450001 Henan China; 3 Key Laboratory of Plant Stress Biology, State Key Laboratory of Cotton Biology, School of Life Sciences, Henan University, Kaifeng, 475001 Henan China; 4 College of Biological Engineering, Henan University of Technology, Zhengzhou, 450001 Henan China; 5 Zhengzhou Tobacco Research Institute, Zhengzhou, 450001 Henan China

**Keywords:** Carotenoid biosynthetic pathway (CBP), Lycopene epsilon-cyclase (ε-LCY), CRISPR/Cas9, High light stress

## Abstract

**Background:**

Lycopene epsilon-cyclase (ε-LCY) is a key enzyme in the carotenoid biosynthetic pathway (CBP) of higher plants. In previous work, we cloned two *Ntε-LCY* genes from allotetraploid tobacco (*Nicotiana tabacum*), *Ntε-LCY2* and *Ntε-LCY1*, and demonstrated the overall effect of *Ntε-LCY* genes on carotenoid biosynthesis and stress resistance. However, their genetic and functional characteristics require further research in polyploid plants.

**Results:**

Here, we used CRISPR/Cas9 to obtain *Ntε-LCY2* and *Ntε-LCY1* mutants in allotetraploid *N.tabacum* K326. *Ntε-LCY2* and *Ntε-LCY1* had similar promoter *cis*-acting elements, including light-responsive elements. The *Ntε-LCY* genes were expressed in roots, stems, leaves, flowers, and young fruit, and their highest expression levels were found in leaves. *Ntε-LCY2* and *Ntε-LCY1* genes responded differently to normal light and high light stress. Both the *Ntε-LCY2* and the *Ntε-LCY1* mutants had a more rapid leaf growth rate, especially *ntε-lcy2-1.* The expression levels of CBP genes were increased in the *ntε-lcy* mutants, and their total carotenoid content was higher. Under both normal light and high light stress, the *ntε-lcy* mutants had higher photosynthetic capacities and heat dissipation levels than the wild type, and this was especially true of *ntε-lcy2-1*. The reactive oxygen species content was lower in leaves of the *ntε-lcy* mutants.

**Conclusion:**

In summary, the expression patterns and biological functions of the *Ntε-LCY* genes *Ntε-LCY1* and *Ntε-LCY2* differed in several respects. The mutation of *Ntε-LCY2* was associated with a greater increase in the content of chlorophyll and various carotenoid components, and it enhanced the stress resistance of tobacco plants under high light.

**Supplementary Information:**

The online version contains supplementary material available at 10.1186/s12870-022-03634-5.

## Background

Solar radiation provides the energy for plant photosynthesis and growth, but photoinhibition may occur when the plant is subjected to excessively high light [1, 2]. Photosystem II (PSII) has long been considered the most sensitive photosynthetic component to high light in plants, and it is the site of both photoinhibition and photooxidation [3, 4]. Under high light intensity when repair processes can not keep pace with the high D_1_ degradation rate, light damage accumulates, exacerbating photoinhibition [5, 6]. Photoinhibition can damage PSII, and this damage increases with higher light intensities and longer stress durations [7]. Therefore, the degree of photoinhibition depends on a balance between PSII photodamage and repair. Photoinhibition also leads to photo-oxidation, which drives the accumulation of excess reactive oxygen species (ROS) in the plant. When ROS levels are too high, protein and lipid peroxidation and even DNA damage may occur [8]. Recent studies have shown that ROS act mainly by inhibiting the repair of damaged PSII [9]. Therefore, plants activate various defense mechanisms to reduce the damage caused by high light stress. For example, carotenoids function in photoprotection, antioxidant processes, and light dissipation under high light [10]. Carotenoids protect photosynthetic organs from ROS-mediated damage by dissipating excess light energy under stress conditions [11]. The carotenoid-based lutein cycle is one mechanism by which plants resist photoinhibition [12]; it can increase the heat dissipation capacity of plants, thereby reducing high light damage [13]. The metabolic balance between carotenoid biosynthesis and catabolism is essential for maintaining appropriate carotenoid content and composition in photosynthetic tissues [14].

Cyclization of the end of the carotenoid C_40_ hydrocarbon chain is an important branch point in the carotenoid biosynthetic pathway (CBP). The lycopene cyclase family is composed of lycopene epsilon*-*cyclase (ε-LCY) and lycopene beta*-*cyclase (β-LCY). The ε-LCY enzyme catalyzes only the formation of a δ ring at one end of the lycopene molecule to generate δ-carotene, and a β ring can then be formed at the other end of δ-carotene through the catalysis of β-LCY. δ-carotene finally forms α-carotene, and α-carotene undergoes hydroxylation and other modifications to form lutein in the α branch of the CBP. The β-LCY enzyme can also catalyze the formation of two β rings at both ends of lycopene to generate β-carotene, which is then hydroxylated and epoxidized to generate β-cryptoxanthin, zeaxanthin, antheraxanthin, violaxanthin, neoxanthin, and other carotenoids in the β branch of the CBP. Therefore, the relative activities of ε-LCY and β-LCY directly determine the ratio of α-carotene to β-carotene substrates, and relative flux through the two branches of the CBP has an important influence on the carotenoid composition of higher plants [15–17]. To date, the *ε-LCY* gene has been cloned from a variety of plants and algae, including *Arabidopsis*, potato, tomato, maize, *Chlorella*, olive, and others [1, 18, 19].

The *ε-LCY* gene exists as a single-copy gene in *Arabidopsis* and maize. It was reported that mutation of *ε-LCY* in *Arabidopsis* increased the contents of β-carotene, violaxanthin, zeaxanthin and other substances, but contents of lutein and other products of the α branch decreased [19]. Downregulation of the *ε-LCY* gene not only increased the carotenoid content of *Brassica napus* seeds [20] but also improved the salt tolerance of sweet potato transgenic callus by increasing the biosynthesis of β branch carotenoids [21]. The expression level of *ε-LCY* can significantly affect carotenoid content and composition in higher plants [22]. Under high temperatures, *LCY* expression determined the ratio of α/β carotene and the content of carotenoids in banana [23]. Mutation of *ε-LCY* in banana increased the β-carotene content approximatelty six-fold in the fruit pulp; the content of α-carotene and lutein were significantly reduced, but agronomic traits were not significantly affected [24]. These research results show that *ε-LCY* plays an important role in the regulation of carotenoid composition and stress resistance in plants, although its specific mechanism of action may differ among plant species.

*N. tabacum* is derived from the hybridization, doubling, and duplication of the ancestral species *Nicotiana sylvestris* and *Nicotiana tomentosiformis*. In *Nicotiana tabacum*, *Ntε-LCY* is present as two highly homologous copies, *Ntε-LCY1* and *Ntε-LCY2*. The overall effect of *Ntε-LCY* gene silencing in tobacco was to improve the total carotenoid and chlorophyll contents while increasing the photosynthetic efficiency [22]. Down-regulation of *Ntε-LCY* gene increased abscisic acid (ABA) levels and enhanced the ability of tobacco to tolerate salt and drought stresses, however, overexpression of *Ntε-LCY* gene reduced the ability of tobacco to tolerate salt and drought stresses [25]. *Ntβ-LCY* overexpression mimicked the phenotype of *Ntε-LCY* gene silencing [26].

The existence of highly homologous genes is a challenging problem for gene functional characterization in polyploid plants. The homologous genes may or may not show strong/weak functional differentiation or temporal and spatial expression differences. Here, we used the model tetraploid plant *N. tabacum* K326 to investigate the functions of two *Ntε-LCY* homologs. We characterized the phenotypes and relevant secondary metabolites of *ntε-lcy* mutants created with CRISPR/Cas9, documented the expression patterns of *Ntε-LCY1* and *Ntε-LCY2*, and analyzed *cis*-elements in their promoters. Finally, we compared the functional characteristics of *Ntε-LCY1* and *Ntε-LCY2* under high light stress.

## Methods

### Plant growth conditions and high light treatment

Seeds of allotetraploid tobacco K326 were provided by the Zhengzhou Tobacco Research Institute. CRISPR/Cas9 mutants of K326 (*ntε-lcy2-1*, *ntε-lcy2-2*, *ntε-lcy1-1*, and *ntε-lcy1-2*) and wild-type (WT) plants were used as the experimental materials. Plants were grown in a greenhouse with a photosynthetic photon flux density (PAR) of 80–250 μmol m^−2^ s^−1^ and a 16-h light/8-h dark photoperiod. The temperature was 25 ± 2 °C, and the relative humidity was 60 ± 2%. Samples used for tissue expression analysis were obtained from 5-month-old wild-type tobacco plants. In other experiments, tobacco plants grown for 45 days were exposed to normal light (PAR = 80–250 μmol m^−2^ s^−1^) or high light (PAR = 440–1000 μmol m^−2^ s^−1^) for 2 h. Tobacco leaf samples in the same part were frozen in liquid nitrogen immediately before use, and additional samples were stored at − 80℃.

### Sequences and bioinformatics analyses of *Ntε-LCY* genes from *N. tabacum*

The coding sequences (CDSs) of the target genes (*Ntε-LCY2* and *Ntε-LCY1*) and of homologous genes from *Nicotiana sylvestris* and *Nicotiana tomentosiformis* were downloaded from the China Tobacco Genome Database v4.0 (http://10.6.0.76/). The CDSs of *Ntε-LCY1* and *Ntε-LCY2* were used as blastn queries to search for *Solanum tuberosum* and *Solanum lycopersicum* homologs in the website (https://phytozome.jgi.doe.gov/). These ε-LCY protein sequences were aligned by ClustalW, the neighbor-joining trees were constructed by using MEGA7 with 1000 replicates of bootstrap [27]. Conserved domains were identified in the predicted *ε-LCY* proteins using MEME 4.12.0 software (parameters: -mod anr -nmotifs 12 -minw 6 -maxw 30) (http://meme-suite.org/tools/meme) [28]. The homology of CDSs were visually inspected with DNAMAN software, and *cis*-acting elements were identified in the 3000-bp promoter regions upstream of *Ntε-LCY1* and *Ntε-LCY2* using PlantCARE (http://bioinformatics.psb.ugent.be/webtools/plantcare/html/) [29]. The I-TASSER program was used to predict the spatial structures, active sites, and ligand-binding sites of Ntε-LCY1 and Ntε-LCY2 proteins (https://zhanglab.ccmb.med.umich.edu/cgi-bin/itasser_submit.cgi) [30].

### Construction of gene editing vectors and identification of homozygous *Ntε-LCY* mutants

The design of target sites and detection primers and the construction of CRISPR/Cas9 vectors for *Ntε-LCY2* and *Ntε-LCY1* were carried out according to the Mutation Sites Based Specific Primers Polymerase Chain Reaction (MSBSP-PCR) method [31]. Exon and intron sequence information for the *Ntε-LCY2* and *Ntε-LCY1* genes was downloaded from the China Tobacco Genome Database, and target sites were identified using CRISPR Multi Targeter (http://www.multicrispr.net/index.html) based on the multiple PAM (NGG or CCN) sites. We selected optimal sgRNA sequences close to the 5' end of the *Ntε-LCY2* and *Ntε-LCY1* CDSs. Appropriate primers were designed ~ 150 bp upstream and downstream of the target site to detect the position and sequence of gene editing in *Ntε-LCY2* and *Ntε-LCY1* transgenic lines (Table S2).

Next, the pSHE401 vector was modified to enable the precise mutation of *Ntε-LCY2* and *Ntε-LCY1* [31]. The *Ntε-LCY2* and *Ntε-LCY1* recombinant vectors were transformed individually into tobacco callus, and T_0_ transgenic-positive plants were identified based on the presence of the kanamycin resistance gene sequence using kanamycin gene primers (Table S2). Their seeds were harvested, T_1_ generation plants were obtained. The mutation sites of the gene-editing positive plants were identified. using the MSBSP-PCR method [31]. The procedure was as follows:If first round of PCR reaction produced amplified products (Primers used: *Ntε-LCY1/2*-F + *Ntε-LCY1/2*-R), and the second round of PCR reaction produced no amplified products (Primers used: *Ntε-LCY1/2*-Target + *Ntε-LCY1/2*-R) (Table S2), and then picked 100 µL of positive bacterial solution and sent them to Beijing Tsingke Biotechnology Co., Ltd. for bacterial solution sequencing verification, and used Geneious software to check the sequence results, Finally *Ntε-LCY1* and *Ntε-LCY2* homozygous mutant lines were identified.

### Phenotypic observations of the* Ntε-LCY* mutants

To minimize the influence of off-target gene editing effects, two separate T_2_ homozygous mutant lines for each gene were used for phenotypic observations and analysis. The maximum length and maximum width of the third true leaf (L3) from the top of WT and *ntε-lcy2-1*, *ntε-lcy2-2*, *ntε-lcy1-1*, and *ntε-lcy1-2* mutant plants were measured with a ruler (12 plants per genotype). The phenotypes of WT, *ntε-lcy2-1*, and *ntε-lcy1-1* plants were photographed.

### Measurement of chlorophyll a and chlorophyll b contents

Chlorophyll a and chlorophyll b contents were measured as described in [32].

### Extraction and quantitative analysis of carotenoids

Fresh, freeze-dried tobacco leaves were ground into powder with a ball mill (30 Hz, 1 min). An appropriate amount of internal standard was added to 50 mg of the ground sample, and carotenoids were extracted with a mixture (1:1:2, v/v/v) of n-hexane, acetone, and ethanol that contained 0.01% BHT (g/mL). The extract was vortexed for 20 min at room temperature. After repeating the extraction, the supernatant was collected by centrifugation, then evaporated to dryness under nitrogen flow and reconstituted in a 3:1 mixture (v/v) of methanol and MTBE. Finally, the solution was filtered through a 0.22-μm filter and analyzed by high performance liquid chromatography-photo diode array detection-mass spectrometry (HPLC–DAD-MS) method with atmospheric pressure chemical ionization (APCI) mode for qualitative [33]. Analyst 1.6.3 software was used to process the mass spectrometry data. The integrated peak area ratios of all samples were entered into the standard curve equation, and the absolute contents of various carotenoids in the actual samples were calculated.

### Total RNA extraction and quantitative real-time PCR (qRT-PCR)

Total RNA was extracted from leaf tissue of *ntε-lcy2-1*, *ntε-lcy1-1*, and WT plants using the Spin Column Plant Total RNA Purification Kit (Shenggong, China), and cDNA was synthesized from the extracted RNA using TransScript One-Step gDNA Removal and cDNA Synthesis SuperMix (Transgen, China), and the RNA/cDNA quality was checked using a ultra-versus spectrophotometer. qRT-PCR was performed using Super Real PreMix Plus (Tiangen, China) on a Light Cycler 480 II system (Roche, Switzerland). The 2^−∆∆Ct^ method was used to calculate relative gene expression [34], using the 26 s RNA gene as an internal reference. The qRT-PCR primers for the *Ntε-LCY* genes and related genes in the CBP were designed with Primer-BLAST software [35] and are listed in Table S3.

### Measurement of chlorophyll fluorescence parameters

Chlorophyll fluorescence parameters were measured using an Imaging-PAM-MAXI chlorophyll fluorescence meter (Walz, Germany) between 9:00 a.m. and 11:00 a.m. The chlorophyll fluorometer was connected to a computer, and ImagingWin software (Walz, Germany) was used for data acquisition. The experimental protocol followed that described in [36].

There were two different groups of tobacco seedlings, one exposed to normal light (PAR = 80 μmol m^−2^ s^−1^), one exposed to high light (PAR = 440 μmol m^−2^ s^−1^) for 2 h. ImagingWin software was used to measure six areas of individual leaves, and five sets of leaves were measured for each genotype. Chlorophyll fluorescence parameters were calculated as follows: maximum photosynthetic efficiency of PSII (Fv/Fm) = (Fm − F_0_)/Fm, and non-photochemical quenching (NPQ) = (Fm-Fm')/Fm' [37].

### Measurement of O_2_^−^ and H_2_O_2_ content

We analyzed the O_2_^−^ content using the nitroblue tetrazolium (NBT) staining method. First, we added 5 mg of NBT to 50 mL 25 mM Hepes buffer solution and then added 50 μL 0.1% Triton X-100 to make a 0.1 mg/mL NBT solution. We used a punch to obtain tobacco leaf discs having a diameter of 0.5 cm from the same part of each plant. We immersed all the samples in the NBT dye solution, stained them for 24 h at 28℃ in the dark, removed the dye solution, added 80% ethanol, and placed the samples in a boiling water bath for 3 min. We removed the 80% ethanol, added absolute ethanol, and kept the samples in the boiling water bath for another 3 min. If necessary, these steps were repeated until no green color remained. We added absolute ethanol to the completely decolorized samples and photographed them under a microscope (Smartzoom5, Zeiss, Germany). H_2_O_2_ content was measured following the manufacturer’s instructions of a hydrogen peroxide kit (Suzhou Comin, China) based on absorbance at 415 nm measured with a microplate reader (Multiskan GO, Thermo Scientific, USA). We confirm that all materials and methods were performed in accordance with the relevant guidelines/regulations/legislation in China.

### Statistical analysis

All experiments included at least three independent technical and biological replicates, and data are expressed as the mean ± standard error of the mean (SEM). All data analyses and processing were performed using Microsoft Excel and GraphPad Prism 5 software (GraphPad Software, Inc. USA). Data for each experiment were compared among WT, *ntε-lcy2-1*, and *ntε-lcy1-1* lines using one- or two-way ANOVA and Tukey’s post-hoc test (*p < 0.05, **p < 0.01, ***p < 0.001).

## Results

### Sequences and bioinformatics analyses of* Ntε-LCY* genes from *N. tabacum*

To investigate the genetic basis for potential functional differentiation between *Ntε-LCY2* and *Ntε-LCY1*, we analyzed their phylogenetic relationships, conserved domains, and homology to related genes in Solanaceae species. MEME analysis of their predicted amino acid sequences showed that Ntε-LCY2 and Ntε-LCY1 were highly conserved and had similarities and differences in their conserved motifs. Both *Ntε-LCY2* and *Ntε-LCY1* contained motifs 9, 5, 1, 8, 11, 10, 12, 2, 4, and 6, but *Ntε-LCY2* contained motif 3 and *Ntε-LCY1* contained motif 7. The CDS of *Ntε-LCY2* is 1497 bp in length and encodes 498 amino acids, whereas that of *Ntε-LCY1* was 1431 bp in length and encoded 476 amino acids. The coding sequence of the *ε-LCY* gene in both ancestral species was 1431 bp in length and encoded 476 amino acids. One *ε-LCY* gene was identified in tomato (*Solanum lycopersicum*, Sly) and potato (*Solanum tuberosum*, Stu). In both cases, its coding sequence was 1584 bp in length and encoded 528 amino acids. The homology of the *Ntε-LCY2* and *Ntε-LCY1* CDSs was 87.95%*.* The phylogenetic relationships, conserved domains, and percent homologies of *Ntε-LCY2*, *Ntε-LCY1*, and related Solanaceae genes are presented in Fig. S1a*.* Although Ntε-LCY2 and Ntε-LCY1 proteins had very similar predicted structures, ligand binding sites, and enzyme active sites, there were some differences. For example, Ntε-LCY2 had a predicted F280 active site in motif 3 and a Y465 active site in motif 6, but these were absent in Ntε-LCY1. These differences may be related to the functional characteristics of Ntε-LCY2 and Ntε-LCY1 proteins (Fig. S1a–b).

The 3000-bp promoter regions of *Ntε-LCY2* and *Ntε-LCY1* contained a number of conserved motifs, such as ARE, MBS, ABRE, and AE-box. The unique motifs in the *Ntε-LCY2* promoter were TGA-element, CA-motif, GA-motif, TCT-motif, chs-CMA1a, and chs-CMA2a. The unique motifs in the *Ntε-LCY1* promoter were TC-rich repeats, HD-Zip1, AT1-motif, ACE, G-box, and LTR. Both promoters contained light response elements, consistent with a potential function in protection against high light stress (Table S1).

### Phenotypes of *Ntε-LCY* mutants generated by CRISPR/Cas9-mediated editing

**Fig. 1:**
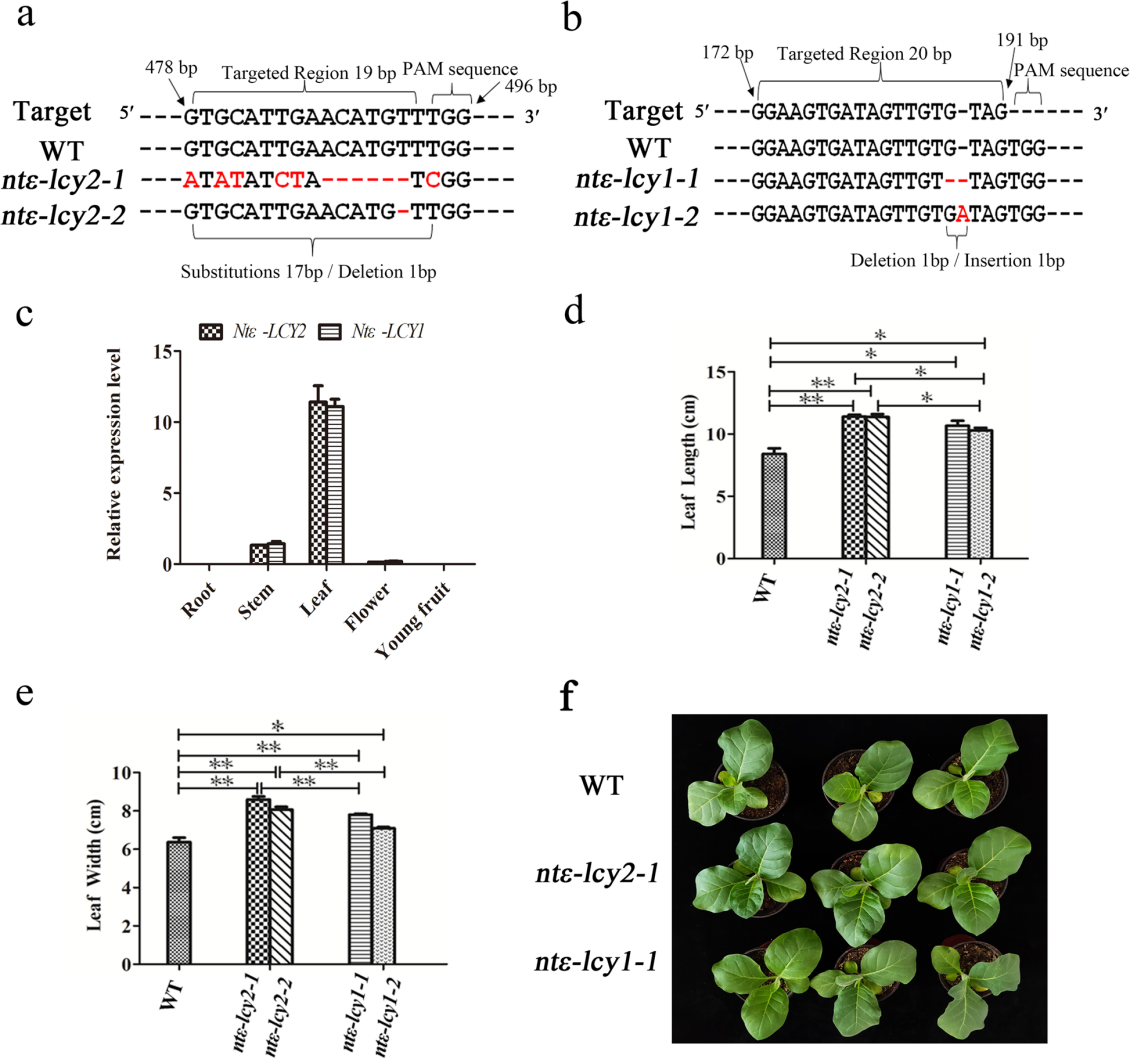
Identification and phenotypic analysis of homozygous *Ntε-LCY* mutants. **a–b** Sequence analysis of *Ntε-LCY2* and *Ntε-LCY1* homozygous mutant strains generated by CRISPR/Cas9; the red font indicates the gene editing area. **c** Tissue expression patterns of *Ntε-LCY2* and *Ntε-LCY1* in roots, stems, leaves, flowers and young fruit by qRT-PCR. Data are presented as the mean ± standard error of the mean (SEM) of three independent experiments (*n* = 3; two-way ANOVA; Tukey’s post-hoc test). **d–e** Maximum length and width of the third true leaf (L3) of five tobacco materials (WT, *ntε-lcy2-1*, *ntε-lcy2-2*, *ntε-lcy1-1*, and *ntε-lcy1-2*). Data are presented as the mean ± SEM of at least 12 plants. Scale bar = 1 cm (*n* = 12 *p < 0.05, **p < 0.01; one-way ANOVA; Tukey’s post-hoc test). **f** Phenotypes of three lines (WT, *ntε-lcy2-1*, and *nt ε-lcy1-1*), and the interval of each two plants is 15 cm

To investigate the functions of the *Ntε-LCY* genes, we used CRISPR/Cas9-mediated editing to generate mutants of *Ntε-LCY2* and *Ntε-LCY1* using the MSBSP-PCR method [31]. Four lines (*ntε-lcy2-1*, *ntε-lcy2-2*, *ntε-lcy1-1*, and *ntε-lcy1-2*) produced amplification products of ~ 300 bp in the first round of PCR and produced no amplification products in the second round, indicating that *Ntε-LCY2* and *Ntε-LCY1* homozygous mutants had been generated successfully (Fig. S2). Corresponding bacterial liquid was successfully sequenced and verified (Fig. S3; Fig. 1a–b). Sequence alignment showed that the target site of *Ntε-LCY2* was located between 478 and 496 bp from the 5′ to the 3′ end*. ntε-lcy2-1* contained a homozygous 17-bp base substitution between 478 and 494 bp, and *ntε-lcy2-2* contained a homozygous 1-bp deletion at 492 bp (Fig. 1a). The target site of *Ntε-LCY1* was between 172 and 191 bp from the 5′ to the 3′ end. *ntε-lcy1-1* contained a homozygous 1-bp deletion at 187 bp, and *ntε-lcy1-2* contained a homozygous 1-bp insertion at 188 bp (Fig. 1b).

*Ntε-LCY2* and *Ntε-LCY1* genes were weakly expressed in roots, stems, flowers, and young fruit; the highest transcript levels were detected in leaves, which were then used for subsequent gene functional analyses (Fig. 1c). Notably, *Ntε-LCY2* and *Ntε-LCY1* had similar expression patterns in all tissues. There was therefore no evidence for temporal or spatial expression differences, although functional differentiation could not be ruled out.

Our previous studies revealed that silencing of *Ntε-LCY* led to higher leaf carotenoid content, and carotenoids have essential roles in plant development. We therefore collected basic phenotypic data on the WT and *Ntε-LCY* mutant lines. At the four-leaf stage, WT, *ntε-lcy2-1*, *ntε-lcy2-2*, *ntε-lcy1-1*, and *ntε-lcy1-2* plants differed significantly in the maximum length and width of the third true leaf (L3). L3 leaf length and width were largest in the *ntε-lcy2* lines, somewhat lower in the *ntε-lcy1* lines, and smallest in the WT plants (Fig. 1d–e). We therefore used leaf tissues from WT, *ntε-lcy2-1*, and *ntε-lcy1-1* plants in subsequent research. When the *ntε-lcy2-1* and *ntε-lcy1-1* mutants had four leaves, the fourth leaves of the WT plants were not always visible. Leaves grew larger and more rapidly in *ntε-lcy2-1*, suggesting that mutation of *Ntε-LCY2* led to more plant benefits (Fig. 1f).

### The accumulation of carotenoids was enhanced in the leaves of* Ntε-LCY* mutants

**Fig. 2:**
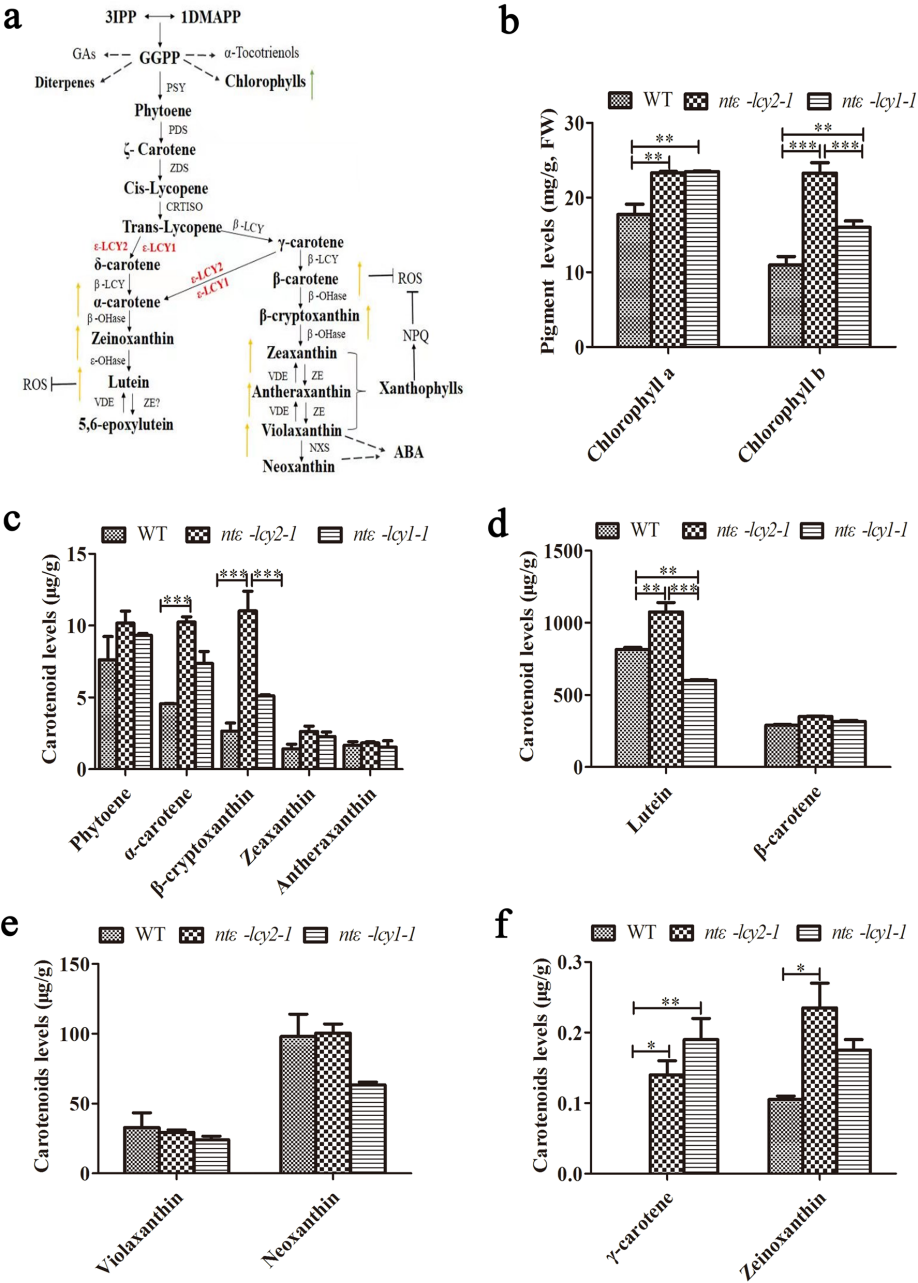
Mutation of *Ntε-LCY* affects the carotenoid and chlorophyll contents of leaves. **a** Diagram of the carotenoid biosynthetic pathway (CBP). Target genes studied in this experiment are printed in red. Isopentenyl diphosphate (IPP), dimethylallyl diphosphate (DMAPP), geranylgeranyl diphosphate (GGPP), phytoene synthase (*PSY*), phytoene desaturase (*PDS*), ζ-carotene desaturase (*ZDS*), carotenoid isomerase (*CRTISO*), *β*-lycopenecyclase (*β-LCY*), *β*-carotene hydroxylase (*β-OHase*), violaxanthin deepoxidase (*VDE*), zeaxanthin epoxidase (*ZE*), and neoxanthin synthase (*NXS*) are shown. **b–f** Relative carotenoid and chlorophyll levels in WT and *Ntε-LCY* mutant strains. Chlorophyll a, Chlorophyll b, phytoene, α-carotene, β-cryptoxanthin, zeaxanthin, antheraxanthin, lutein, β-carotene, violaxanthin, neoxanthin, γ-carotene, and zeinoxanthin are shown. Data are presented as the mean ± SEM of three independent experiments (*n* = 3, *p < 0.05, **p < 0.01, ***p < 0.001; two-way ANOVA, Tukey’s post-hoc test)

To better understand the effect of *Ntε-LCY* mutation on the CBP (Fig. 2a) and on the leaf phenotype of *Ntε-LCY* mutants, we examined the content of different carotenoids in the *ntε-lcy2-1* and *ntε-lcy1-1* mutants by high performance liquid chromatography-photo diode array detection-mass spectrometry (HPLC–DAD-MS) method with atmospheric pressure chemical ionization (APCI) mode [33]. The *ntε-lcy2-1* mutants showed the highest carotenoid levels, followed by the *ntε-lcy1-1* mutants and the WT plants, consistent with the leaf phenotype data. Among the main types of carotenoids, α-carotene and β-cryptoxanthin contents were significantly higher in the *Ntε-LCY* mutants, especially in the leaves of *ntε-lcy2-1* (Fig. 2c). The contents of phytoene, zeaxanthin, and β-carotene were also somewhat higher in the *Ntε-LCY* mutants (Fig. 2c–d). The lutein content of *ntε-lcy2-1* was significantly higher than that of *ntε-lcy1-1* and WT (Fig. 2d), and the zeinoxanthin content was clearly higher in the *Ntε-LCY* mutants than in the WT (Fig. 2f). There were no significant differences in antheraxanthin or violaxanthin content among the genotypes (Fig. 2c–e).

Chlorophyll a (Chl a) and Chlorophyll b (Chl b) contents were also much higher in the *Ntε-LCY* mutants. Chlorophyll content showed the same trend as leaf growth and carotenoid content: highest in *ntε-lcy2-1*, followed by *ntε-lcy1-1* and the WT. Together, these data suggested that mutation of *Ntε-LCY* genes indeed enhanced the accumulation of carotenoids and chlorophylls. *Ntε-LCY2* mutation promoted carotenoid and chlorophyll accumulation to a greater extent than *Ntε-LCY1* mutation, suggesting that the homologs exhibited strong and weak functional differentiation.

### *Ntε-LCY2* mutation has stronger effects than *Ntε-LCY1* mutation on carotenoid biosynthesis

**Fig. 3:**
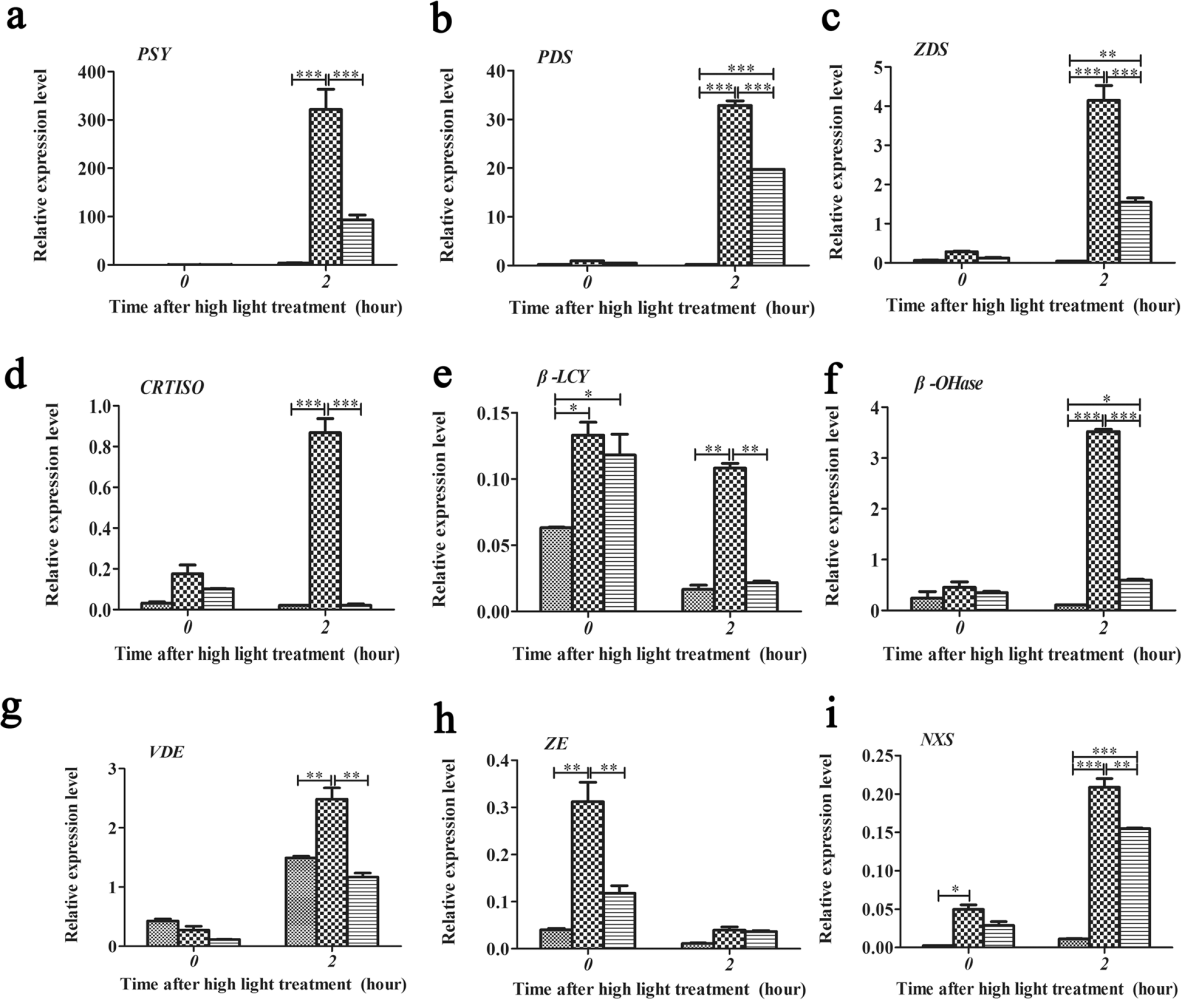
Relative expression of upstream and downstream genes related to the *Ntε-LCY* branch of the CBP after exposuring to normal light (photon flux density (PAR) = 250 μmoL m^−2^ s^−1^) and high light ( photon flux density (PAR) = 1000 μmoL m^−2^ s^−1^) for 2 h. Phytoene synthase (*PSY*), phytoene desaturase (*PDS*), ζ-carotene desaturase (*ZDS*), carotenoid isomerase (*CRTISO*), *β*-lycopenecyclase (*β-LCY*), *β*-carotene hydroxylase (*β-OHase*), violaxanthin deepoxidase (*VDE*), zeaxanthin epoxidase (*ZE*), and neoxanthin synthase (*NXS*) are shown. Data are presented as the mean ± SEM of three independent experiments (*n* = 3, *p < 0.05, **p < 0.01, ***p < 0.001; two-way ANOVA, Tukey’s post-hoc test)

The products of most CBP enzymes were present at higher levels in the *ntε-lcy2-1* and *ntε-lcy1-1* mutants. The first CBP product, phytoene, is crucial for the regulation of the entire pathway. However, the contents of lutein and β-carotene, the main carotenoid components in *N. tabacum*, were also clearly increased in the mutants*.* We next examined the expression levels of genes that encoded CBP enzymes. qRT-PCR results showed that the expression levels of phytoene synthase (*PSY*), phytoene desaturase (*PDS*), ζ-carotene desaturase (*ZDS*), carotenoid isomerase (*CRTISO*), *β*-lycopene cyclase (*β-LCY*), *β*-carotene hydroxylase (*β-OHase*), violaxanthin deepoxidase (*VDE*), zeaxanthin epoxidase (*ZE*), and neoxanthin synthase (*NXS*) genes were upregulated in *ntε-lcy* plants under normal growing conditions. Their expression patterns showed the same trend: highest in *ntε-lcy2-1*, followed by *ntε-lcy1-1*, and lowest in the WT. Notably, the expression levels of *β-LCY*, *ZE*, and *NXS* were significantly higher in *ntε-lcy* plants than in the WT. However, the expression level of *VDE* was lower in *Ntε-LCY* mutants than in the WT under normal conditions (Fig. 3).

Carotenoids can protect the photosystems from photodamage and photoinhibition under high light stress. To gain insights into the biological effects of *Ntε-LCY* mutation, we used qRT-PCR to examine the expression of CBP enzyme genes in WT, *ntε-lcy2-1*, and *ntε-lcy1-1* after a 2 h exposure to high light. Under high light stress, the relative expression levels of *PSY*, *PDS*, *ZDS*, *β-OHase*, *VDE*, and *NXS* were strongly upregulated in *ntε-lcy* mutants compared to their expression under normal growing conditions. The genotypes showed the same trend from high (*ntε-lcy2-1*) to low (WT). By contrast, the relative expression of *CRTISO* was significantly upregulated in *ntε-lcy2-1* but downregulated in *ntε-lcy1-1* and the WT. The relative expression of *β-LCY* was significantly downregulated in *ntε-lcy1-1* and WT plants but only slightly affected by high light stress in *ntε-lcy2-1*. Finally, the relative expression of *ZE* was downregulated in the *Ntε-LCY* mutants and the WT under high light stress. Together, these results demonstrate that *Ntε-LCY2* mutation produces stronger effects than *Ntε-LCY1* mutation on carotenoid biosynthesis and the photosynthetic system*.*

### *Ntε-LCY2* and *Ntε-LCY1* genes showed different light response patterns

**Fig. 4:**
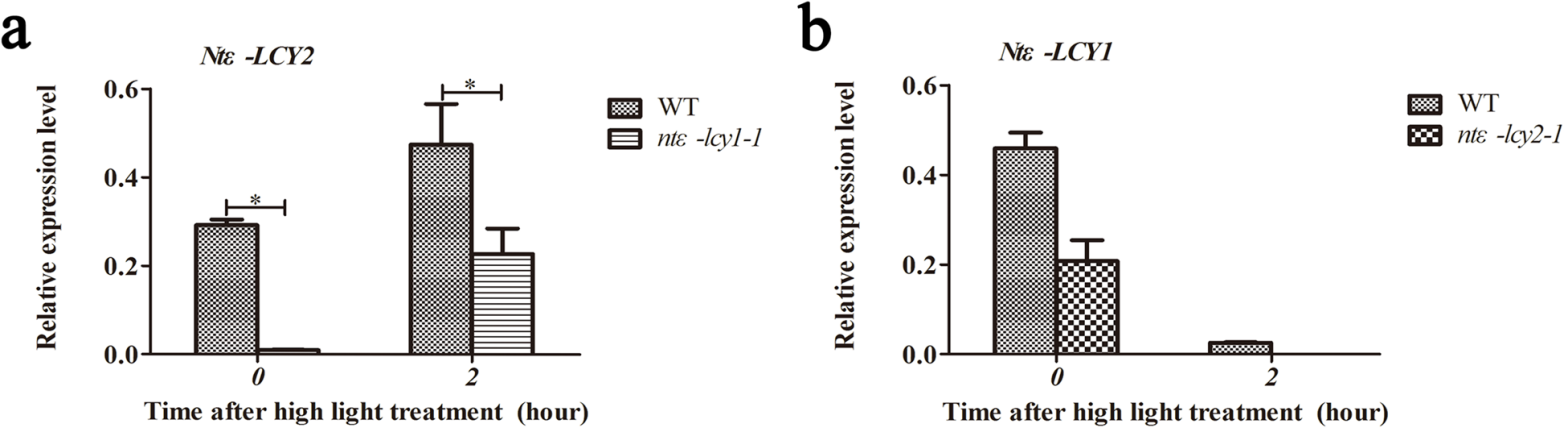
*Ntε-LCY2* and *Ntε-LCY1* genes have different light response patterns in leaves. **a** Response pattern of *Ntε-LCY2* in WT and *ntε-lcy1-1* lines after exposure to normal light (PAR = 250 μmoL m^−2^ s^−1^) and high light (PAR = 1000 μmoL m^−2^ s^−1^) for 2 h measured by qRT-PCR. **b** Response pattern of *Ntε-LCY1* in WT and *ntε-lcy2-1* lines after exposuring to normal light (PAR = 250 μmoL m^−2^ s^−1^) and high light (PAR = 1000 μmoL m^−2^ s^−1^) for 2 h measured by qRT-PCR. (*n* = 3, *p < 0.05; two-way ANOVA, Tukey’s post-hoc test)

Given that *Ntε-LCY* mutation could strongly induce carotenoid-related gene expression and increase carotenoid and chlorophyll levels, we further analyzed the expression of the two *Ntε-LCY* genes under normal light and high light stress using qRT-PCR. Under normal light conditions, the relative expression of *Ntε-LCY2* was significantly higher in WT than in *ntε-lcy1-1*. In fact, the relative expression of *Ntε-LCY2* in *ntε-lcy1-1* was almost zero. After 2 h of high light exposure, the expression of *Ntε-LCY2* was upregulated in both WT and *ntε-lcy1-1*, but its expression was still significantly higher in WT. This result indicated that the expression of *Ntε-LCY2* is inhibited in the *ntε-lcy1-1* mutant and that high light induces *Ntε-LCY2* expression (Fig. 4a). However, high light exposure repressed the expression of *Ntε-LCY1*, and *Ntε-LCY1* expression was also inhibited in the *ntε-lcy2-1* mutant (Fig. 4b). Under normal light conditions, the relative expression of *Ntε-LCY1* was higher in WT than in *ntε-lcy2-1*. After 2 h of high light exposure, the relative expression of *Ntε-LCY1* was downregulated in both WT and *ntε-lcy2-1* (Fig. 4b). Therefore, expression of *Ntε-LCY1* or *Ntε-LCY2* was repressed by the mutation of its homolog. The two genes also showed contrasting responses to high light stress: high light induced *Ntε-LCY2* expression but repressed *Ntε-LCY1* expression, perhaps owing to differences in their promoter *cis*-elements. These results suggested that *Ntε-LCY2* may have a more important function in α-carotene biosynthesis, as both *Ntε-LCY2* transcripts and lutein accumulated to a greater extent in response to high light stress (Fig. 4).

### The photosynthetic apparatus was protected in *Ntε-LCY* mutants under high light stress, especially in *ntε-lcy2*

**Fig. 5:**
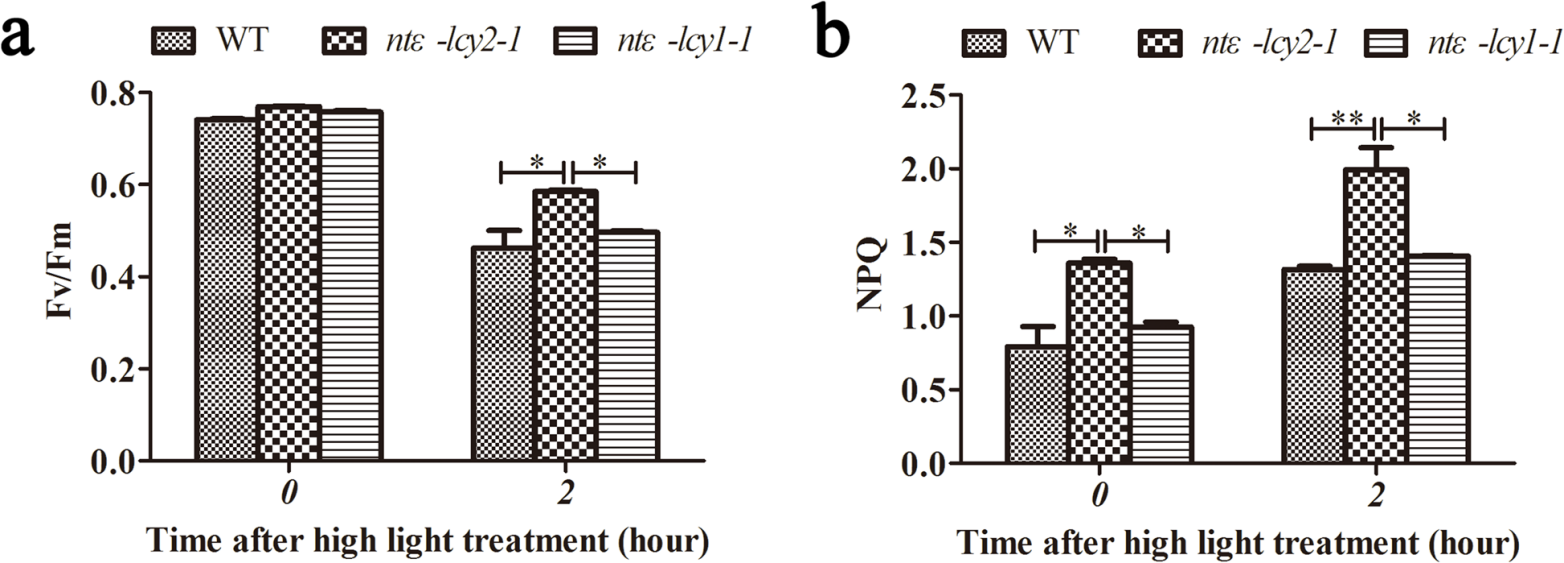
Effect of high light stress on photosynthetic parameters of detached leaves of *Ntε-LCY* mutant plants. **a** Effect of a 2-h exposure to normal light or high light stress on the leaf maximum photochemical efficiency of PSII (Fv/Fm) in *Ntε-LCY* mutant plants. **b** Non-photochemical quenching of chlorophyll fluorescence (NPQ) in detached leaves of *Ntε-LCY* mutant plants exposed to high light stress for 2 h. The photon flux density (PAR) of normal light was 80 μmoL m^−2^ s^−1^, and that of high light was 440 μmoL m^−2^ s^−1^. Data are presented as the mean ± SEM of four independent experiments (*n* = 4, *p < 0.05, **p < 0.01; two-way ANOVA, Tukey’s post-hoc test)

To gain insight into the biological effects of *Ntε-LCY* mutation, we examined the photosynthetic parameters of 45-day-old WT, *ntε-lcy2-1*, and *ntε-lcy1-1* seedlings after exposuring to high light stress for 2 h. Under normal light conditions, the maximum photochemical efficiency of PSII (Fv/Fm) was significantly higher in *Ntε-LCY* mutant plants than in the WT. However, there was no clear difference in Fv/Fm between *ntε-lcy2-1* and *ntε-lcy1-1*. After a 2 h exposure to high light stress, Fv/Fm was lower in all genotypes, but it was higher in the *Ntε-LCY* mutant plants than in the WT plants, especially in the *ntε-lcy2-1* mutant (Fig. 5a).

We next examined non-photochemical quenching (NPQ) in 45-day-old WT, *ntε-lcy2-1*, and *ntε-lcy1-1* seedlings after exposuring to high light stress for 2 h (Fig. 5b). Under normal light conditions, NPQ was higher in the *Ntε-LCY* mutant plants than in the WT plants, and NPQ was significantly higher in *ntε-lcy2-1* plants than in WT and *ntε-lcy1-1* plants. After a 2 h exposure to high light stress, NPQ increased significantly in all genotypes; it was the highest in the *ntε-lcy2-1* plants, followed by *ntε-lcy1-1* and WT plants. These data indicated that photosynthetic capacity and heat dissipation capacity were higher in *Ntε-LCY* mutant plants than in WT plants regardless of light level, consistent with their enhanced accumulation of carotenoids and chlorophylls.*Ntε-LCY2* mutation promoted the accumulation of carotenoids in the β-carotene branch of the CBP, thereby promoting light stress adaptation. In particular, lutein wss essential for NPQ, and the higher lutein content of *ntε-lcy2-1* can be seen in Fig. 2d. Together, these results showed that *Ntε-LCY* mutations, especially mutation of *Ntε-LCY2*, directed the metabolic flux toward β-carotene biosynthesis and improved photosynthetic efficiency in tetraploid *N. tabacum*.

### Mutation of *Ntε-LCY2* and *Ntε-LCY1* reduced ROS accumulation in tobacco leaves under normal and high light stress conditions

**Fig. 6:**
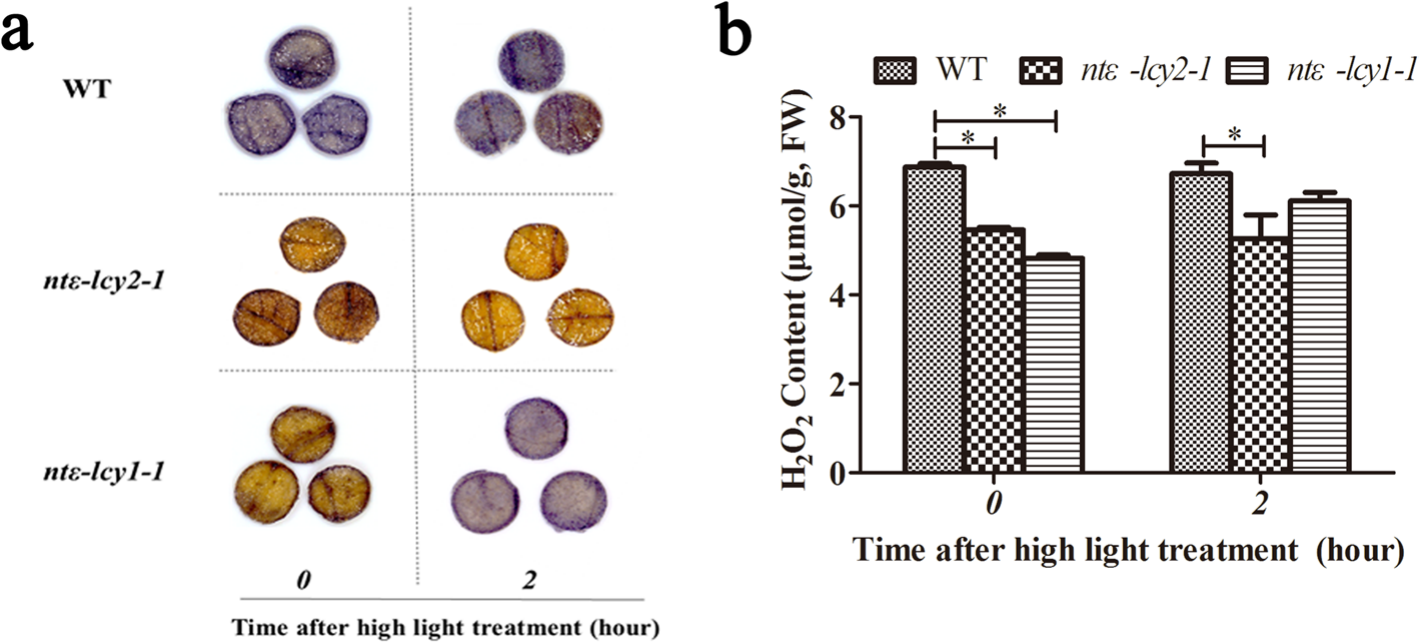
The mutation of *Ntε-LCY* genes reduced the accumulation of reactive oxygen species in leaves to different extents after exposure to normal light or high light stress in WT, *ntε-lcy2-1*, and *ntε-lcy1-1* lines. **a** In situ O_2_^−^ production in 1-cm leaf discs by NBT staining (*n* = 12). **b** Total H_2_O_2_ content of WT and *ntε-lcy* lines after exposuring to normal light or high light stress. Data are presented as the mean ± SEM of three independent experiments (*n* = 3, *p < 0.05; two-way ANOVA, Tukey’s post-hoc test)

Given the higher carotenoid accumulation and photosynthetic efficiency of the *ntε-lcy* mutants, we next examined their O_2_^−^ and hydrogen peroxide (H_2_O_2_) contents after a 2 h exposure to high light stress. Both *ntε-lcy2-1* and *ntε-lcy1-1* showed lighter nitroblue tetrazolium staining than the WT under normal light conditions, and the degree of staining did not differ significantly between *ntε-lcy2-1* and *ntε-lcy1-1*. After a 2 h exposure to high light, the depth of blue staining was highest in the WT, followed by *ntε-lcy1-1* and *ntε-lcy2-1* (Fig. 6a). Mutation of *Ntε-LCY2* and *Ntε-LCY1* genes reduced the *accumulation* of O_2_^−^ in tobacco leaves under normal and high light conditions.

Similarly, the H_2_O_2_ content was lower in *ntε-lcy2-1* and *ntε-lcy1-1* than in WT under normal light conditions, and it was lowest in *ntε-lcy1-1*. However, after a 2 h high light exposure, H_2_O_2_ content was highest in WT, followed by *ntε-lcy1-1* and *ntε-lcy2-1* (Fig. 6b). H_2_O_2_ accumulation did not change significantly in *ntε-lcy2-1* or WT under high light, but it increased significantly in *ntε-lcy1-1* (Fig. 6b). These results suggested that *Ntε-LCY2* and *Ntε-LCY1* mutation reduced the production and/or enhanced the scavenging of H_2_O_2_ in tobacco leaves under normal and high light conditions. Overall, the mutation of *Ntε-LCY2* and, to a lesser extent, *Ntε-LCY1* increased carotenoid accumulation, improved the maximum efficiency of PSII and NPQ, and reduced the accumulation of ROS.

## Discussion

Homologous genes with high sequence similarity are always present in the genomes of polyploid plants and may show similar or contrasting expression patterns. Owing to gene sequence variation and genome recombination, the functions of homologous genes may change through differentiation, silencing, gain of new function, etc. They may exhibit functional differentiation, strong and weak functional differentiation, and/or spatiotemporal expression differences. During molecular breeding, it is crucial to precisely delineate the functional differentiation of homologs so that the more effective gene can be selected. *ε-LCY* is located at the branch point of the α and β branches of the CBP and therefore directly determines the ratio of α-carotene to β-carotene. There are two *ε-LCY* genes in the *N. tabacum* K326 genome, *Ntε-LCY1* and *Ntε-LCY2*. The two genes had similar expression patterns in different tissues; both had the highest expression in leaves (Fig. 1c), suggesting that they functioned in tobacco leaf growth and development, consistent with the findings of Shi et al. [22]. There were four points of difference between the two homologs. First, bioinformatic predictions indicated that Ntε-LCY1 and Ntε-LCY2 had two different amino acids in the enzyme active site, which may contribute to differences in their enzyme activities (Fig. S1a–b). Second, their promoters contained some unique *cis*-elements (Table S1). Third, high light stress induced the expression of *Ntε-LCY2* but repressed the expression of *Ntε-LCY1*, perhaps owing to their different promoter *cis*-elements. Fourth, *Ntε-LCY2* mutation produced stronger effects than *Ntε-LCY1* mutation on the regulation of carotenoid biosynthesis and photosynthetic parameters. Together, these differences between *Ntε-LCY2* and *Ntε-LCY1* were consistent with classical strong and weak functional differentiation.

The biological function of *ε-LCY* is different from that of other genes in the CBP [20, 22, 24]. Mutation of *ε-OHase* in *Arabidopsis* caused a decrease in lutein content; β-carotene content did not increase significantly, but the contents of violaxanthin and zeaxanthin, which are located downstream of β-carotene, did increase [26]. The *CRTISO* mutant in *Arabidopsis* accumulated large amounts of lycopene and had lower contents of lutein, β-carotene, violaxanthin, and zeaxanthin. Chlorophyll a and chlorophyll b contents were also lower, and the content of violaxanthin decreased slightly [38]. Mutation of *β-OHase* in *Arabidopsis* caused a decrease in lutein, β-carotene, violaxanthin, and zeaxanthin and an increase in α-carotene content [39]. Unlike the former genes, the silencing of *PSY*, *PDS*, *β-LCY*, and *VDE* caused the photobleaching of different plant organs and/or significant declines in photosynthetic efficiency and stress resistance [40–45].Therefore, it would be helpful to study the functional differentiation of homologous genes in allotetraploid tobacco.

The overall effect of downregulating or silencing tobacco *Ntε-LCY* is to increase total carotenoid and chlorophyll contents, photosynthetic efficiency, and levels of the stress response hormone ABA. *Ntε-LCY* silencing can also enhance plant tolerance to salt, drought, and other environmental stresses [22, 25]. Our results showed that the mutation of *Ntε-LCY2* and *Ntε-LCY1* increased the chlorophyll (Fig. 2b) and carotenoid (Fig. 2c–f) contents, decreased photoinhibition (Fig. 5), and reduced the accumulation of ROS (Fig. 6). Therefore, the tolerance of tobacco plants to high light stress appeared to be improved. *Ntε-LCY2* and *Ntε-LCY1* mutation increased the contents of chlorophyll a, chlorophyll b, α-carotene, β-carotene, β-cryptoxanthin, and lutein to varying degrees (Fig. 2b–d). The contents of β-cryptoxanthin and lutein were up to 2.15 times and 1.79 times higher in *ntε-lcy2-1* than in *ntε-lcy1-1* (Fig. 2c–d). Likewise, a previous study in banana reported that β-carotene content increased up to six-fold when *ε-LCY* was edited, but the α-carotene and lutein contents were decreased [24]. NPQ reflects the photoprotective ability of plants; it dissipates excess energy from the photosynthetic electron transport chain and reduces ROS accumulation [46]. Xanthophylls and lutein play an important role in the photoprotection of PSII [47]. *Ntε-LCY2* mutation increased plant carotenoid accumulation, thereby protecting the photosynthetic apparatus and reducing ROS accumulation to a greater extent than *Ntε-LCY1* mutation.

Our results demonstrated that the mutation of *Ntε-LCY2* or its homologs in other plants can be conveniently achieved through CRISPR/Cas9-mediated genome editing or other mutagenesis technology.

## Supplementary Information

**Additional file 1.** Ntε-LCY1 and Ntε-LCY2 related sequences.

**Additional file 2: Figure S1.** Sequences and phylogenetic analysis of thelycopene epsilon-cyclase genes in *Nicotianatobacum*. **a** Phylogenetic tree andconserved domain analysis of amino acid sequences from Solanaceae species. *N. tabacum *(*Ntε-LCY1*), *N. tabacum* (*Ntε-LCY2*),* Nicotianasylvestris* (*Nsyε-LCY*),* Nicotiana tomentosiformis* (*Ntomε-LCY*),* Solanum tuberosum *(*Stuε-LCY*),*and Solanum lycopersicum *(*Slyε-LCY*) sequences are shown. **b** Spatial structures, ligand binding sites, andenzyme active sites of Ntε-LCY2 (497 amino acids) and Ntε-LCY1 (475 amino acids) from* N.tobacum*predicted with the I-TASSER program. **Figure S2.** Screening gelelectrophoresis of homozygous mutants of *Ntε-LCY2*and *Ntε-LCY1*. **a** *ntε-lcy2-1* homozygousmutant line with two rounds of PCR verification. **b** *ntε-lcy2-2* homozygousmutant line with two rounds of PCR verification. **c** *ntε-lcy1-1* homozygousmutant line with two rounds of PCR verification. **d** *ntε-lcy1-2* homozygousmutant line with two rounds of PCR verification. M, DL2000 DNA marker. **FigureS3.** Sequencing verification results of bacterial liquidcorresponding to two rounds of PCR reactions for *Ntε-LCY2* and *Ntε-LCY1*homozygous mutant lines. **a** Partialpeak diagram of WT and two homozygous mutant linesof *Ntε-LCY2* (*ntε-lcy2-1*, *ntε-lcy2-2*) **b** Partial peak diagram of WT and twohomozygous mutant lines of *Ntε-LCY1* (*ntε-lcy1-1*, *ntε-lcy1-2*).** Table S1.** *cis*-acting elementsin *Ntε-LCY* gene promoters (3000 bpupstream of the start codon) identified using PlantCARE online software.Important motifs and their functions are shown to highlight similarities anddifferences among the *Ntε-LCY*homologs. **Table S2**. The primers and targetsequences used to construct the CRISPR/Cas9 vectors for *Ntε-LCY2 *and* Ntε-LCY1* and identify the resulting mutants. **Table S3**. The primers used for quantitative real-time PCR(qRT-PCR) analysis.

## Data Availability

Sequence data of the genes described in this article can be found in supplementary information files, The authors affirm that all data generated or analysed during this study are included in this published article [and its supplementary information files].

## Abbreviations

CBP: Carotenoid biosynthetic pathway
CDSs: Coding sequences
PSII: Photosystem II
Fv/Fm: Maximum photosynthetic efficiency of PSII
PAR: Photosynthetic photon flux density
NPQ: Non-photochemical quenching
NBT: Nitroblue tetrazolium
ROS: reactive oxygen species
H_2_O_2_: Hydrogen peroxide
ABA: Abscisic acid
*Nsyε-LCY*: *Nicotiana sylvestris*
*Ntomε-LCY*: *Nicotiana tomentosiformis*
*Stuε-LCY*: *Solanum tuberosum*
*Slyε-LCY*: *Solanum lycopersicum*
qRT-PCR: Quantitative real-time PCR
IPP: Isopentenyl diphosphate
DMAPP: Dimethylallyl diphosphate
GGPP: Geranylgeranyl diphosphate
*PSY*: Phytoene synthase
*PDS*: Phytoene desaturase
*ZDS*: ζ-Carotene desaturase
*CRTISO*: Carotenoid isomerase
*ε-LCY*: Lycopene epsilon-cyclase
*β-LCY*: Lycopene beta*-*cyclase
*β-OHase*: *β*-Carotene hydroxylase
*VDE*: Violaxanthin deepoxidase
ZE: Zeaxanthin epoxidase
NXS: neoxanthin synthase
