# Supplementary material for: Functional characterization and comparison of lycopene epsilon-cyclase genes in Nicotiana tabacum
Source: BMC Plant Biol. 2022 May 21;22:252. doi: 10.1186/s12870-022-03634-5 (PMC9123772; doi:10.1186/s12870-022-03634-5)
Supplement: Supplementary file 1 — Additional file 1. Ntε-LCY1 and Ntε-LCY2 related sequences. [file 12870_2022_3634_MOESM1_ESM.docx]

>Ntab0006110.1 Nicotiana tabacum CDS sequence（*Ntε-LCY1*）, expressed protein, similar to Lycopene epsilon cyclase, chloroplastic OS=Solanum lycopersicum GN=CRTL-E-1 PE=2 SV=1

ATGGAGTGTATTGGAGCTCGAAATTTTTCTACAATGGCGGTTTTTACGTGTCCGAGATTCAAATCATTAGGAAGAAAGAGAATTATGCCAAGAAAAAAGCAACCATTTTGGCCTATACATATGAAAGTGAAGTGTAGTGGAAGTGATAGTTGTGTAGTGGTTAAAGAAGATTTTGCTGATGAAGAAGATTATATAAAAGCTGGTGGTTCAGAACTTGTTTTTGTTCAAATGCAGCAGAATAAAGACATGGATCTACAGTCTAAGCTTTCTGATAAGTTGCGACAAATATCATCAGCTGGACAAACTATACTGGATTTAGTGGTCATTGGCTGTGGTCCTGCTGGTCTTGCTCTTGCTGCGGAGTCTGCTAAACTCGGGTTGAACGTGGGGCTCGTTGGTCCTGATCTTCCTTTCACAAATAACTATGGTGTCTGGGAAGACGAGTTCAAAGATCTTGGGCTTCAAGCATGCATTGAACATGTTTGGAGTGATACCATTGTATATCTTGATGATGCCGATCCAATTCTTATTGGACGTGCTTATGGAAGAGTTAGTCGCCATTTACTGCATGAGGAGTTACTCAAAAGGTGTGTGGAGGCAGGTGTTTTATATCTTAACTCGAAAGTGGATAGGATCGTTGAGTCCACAAGTGGCCACAGTCTTGTAGAGTGCGAGGGCGACATTGTCATTCCTTGCAGGTTTGTCACTGTTGCATCTGGAGCCGCCTCAGGGAAATTCTTGCAGTATGAGTTGGGAGGTCCTCGGGTTTCTGTTCAAACAGCTTATGGAGTGGAAGTTGAGGAAACTTGTTTGGCTTCAAAAGATGCAATGCCATTTGATCTATTAAAGAAAAAACTGATGTTACGATTGAACACATTGGGCATAAAAATTAAAAAAATCTACGAGGAGGAATGGTCTTACATACCAGTTGGTGGATCGTTGCCAAATACAGAGCAGAAAACACTTGCGTTTGGCGCTGCTGCTAGCATGGTTCATCCAGCTACAGGTTATTCAGTTGTCAGATCACTGTCCGAGGCGCCAAAATGCGCCTCCGTACTTGCAAATATTTTAAGACAAAATCATGTCAAGAACATGATTACCAGTTCAAGTGCCACAAGTATCTCAACTCAAGCTTGGAACACCCTTTGGCCACAAGAACGAAAAAGGCAACGATCGTTTTTCCTATTTGGATTGGCACTCATATTGCAGCTGGATATTGAGGGGATTAGGTCATTTTTCCGCGCATTCTTCCGTGTACCAAAATGGATGTGGCAAGGTTTTCTTGGCTCTAGTCTTTCATCAGCAGACCTCATGTTATTTGCCTTCTACATGTTTATTATTGCACCAAATGACATGAGAAAAGGCCTAATCAGACATCTGTTATCTGATCCAACTGGTGCAACTATGATAAGAACTTATCTTACATTTTAG

>Ntab0455950.1 Nicotiana tabacum CDS sequence （*Ntε-LCY2*）, expressed protein, similar to Lycopene epsilon cyclase, chloroplastic OS=Solanum lycopersicum GN=CRTL-E-1 PE=2 SV=1

ATGGATTGTATTGGAGCTCGAAATTTTGCTACAATGGCGGTTTTTACGTGTCCGAGATTCAAATCATTAGGAAGAAGGAGAATTATGCCAAGAAAAAAGCAACCAATTTGGCCTATACATATGCAAGTGAAGTGTAGTGGAAATGAGAGTTGTGTAGTAGTTAAAGAAGATTTTGCCGATGAAGAGGATTATATAAAAGCTGGTGGTTCAGAACTTGTTTTTGTTCAAATGCAGCAGAATAAAGACATGGATCTGCAGTCTAAGCTTTCTGATAAGTTGCGACAAATATCATCAGCTGGACAAACTATACTGGATTTGGTGGTCATAGGCTGTGGTCCTGCTGGTCTTGCTCTTGCTGCGGAGTCTGCTAAACTCGGATTGAACGTTGGGCTCGTTGGTCCTGATCTTCCTTTCACAAATAACTATGGTGTTTGGGAGGATGAGTTCAAAGATCTTGGGCTTCAAGCGTGCATTGAACATGTTTGGAGGGATACCATAGTATATCTTGACGATGCCGATCCAATTCTTATCGGTCGTGCTTATGGAAGAGTTAGTCGCCATTTACTGCACGAGGAGTTACTCAAAAGGTGTGTGGAGGCAGGTGTTTTATATCTTAACTCGAAAGTGGATAGGATCGTTGAGTCCACAAGTGGCCACAGTCTTGTAGAGTGCGAGGGCGACATTGTCATTCCTTGCAGGTTTGTCACTGTTGCATCTGGTGCTGCCTCAGGGAAATTCTTGCAGTATGAGTTGGGAGGTCCTCGGGTTTCTGTTCAAACAGCTTATGGAGTGGAAGTTGAGGTCGATAACAATCCGTATGATCCAAGCCTGATGGTTTTCATGGATTATAGAGACTATGTCAGACACGAAACTTGTTTGGCTTCAAAAGATGCAATGCCATTTGATTTGTTAAAGAAAAAACTGATGTTACGATTGAACACACTGGGTGTAAGAATTAAGCAAATCTACGAGGAGGAATGGTCATACATACCAGTTGGTGGATCTTTACCAAATACCGAGCAAAAAACACTTGCATTTGGTGCTGCTGCTAGCATGGTTCATCCAGCTACAGGTTATTCAGTTGTCAGATCACTGTCCGAGGCACCAAAATGCGCCTCCGTACTTGCTAATATTTTACGACAAAATCATGTCAAGAACATGCTAACCAGTTCAAGTACCACAAGTATCTCAACTCAAGCTTGGAACACCCTTTGGCCACAAGAACGAAAAAGGCAACGATCGTTTTTCCTATTTGGATTGGCACTCATATTGCAGTTGGATATTGAGGGGATTAGGTCATTTTTCCGCGCATTCTTCCGTGTGCCAAAATGGATGTGGCAAGGATTTCTTGGCTCTAGTCTTTCATCAGCAGACCTCATGTTATTTGCCTTCTACATGTTTATTATTGCACCAAATGACATGAGAAAAGGCCTAATCAGACATTTGTTATCTGATCCAACTGGTGCAACCATGATAAGAACTTATCTTACATTTTAG

>lcl|Ntom0117240.1 expressed protein （*Ntomε-LCY*）, similar to Lycopene epsilon cyclase, chloroplastic OS=Solanum lycopersicum GN=CRTL-E-1 PE=2 SV=1

ATGGAGTGTATTGGAGCTCGAAATTTTGCTACAATGGCGGTTTTTACGTGTCCGAGATTCAAATCATTAGGAAGAAGGAGAATTATGCCAAGAAAAAAGCAACCATTTTGGCCTATACATATGCAAGTGAAGTGTAGTGGAAATGAGAGTTGTGTAGTAGTTAAAGAAGATTTTGCTGATGAAGAGGATTATATAAAAGCTGGTGGTTCAGAACTTGTTTTTGTTCAAATGCAGCAGAATAAAGACATGGATCTGCAGTCTAAGCTTTCTGATAAGTTGCGACAAATATCATCAACTGGACAAACTATACTGGATTTGGTGGTCATAGGTTGTGGTCCTGCTGGTCTTGCTCTTGCCGCGGAGTCTGCTAAACTCGGATTGAACGTTGGGCTCGTTGGTCCTGATCTTCCTTTCACAAATAACTATGGTGTTTGGGAGGATGAGTTCAAAGATCTTGGACTTCAAGCGTGCATTGAACATGTATGGAGGGATACCATTGTATATCTTGACGATGCCGATCCAATTCTTATCGGTCGTGCTTATGGAAGAGTTAGTCGCCATTTACTGCACGAGGAGTTACTCAAAAGGTGTGTGGAGGCAGGTGTTTTATATCTTAACTCGAAAGTGGATAGGATCGTTGAGTCCACAAGTGGCCACAGTCTTGTAGAGTGCGAGGGCGACATTGTCATTCCTTGCAGGTTTGTCACTGTTGCATCTGGAGCCGCCTCAGGGAAATTCTTGCAGTATGAGTTGGGAGGTCCTCGGGTTTCTGTTCAAACAGCTTATGGAGTGGAAGTTGAGGAAACTTGTTTGGCTTCAAAAGATGCAATGCCATTTGATTTGTTAAAGAAAAAGCTGATGTTACGATTGAACACACTGGGTGTAAGAATTAAGCAAATCTACGAGGAGGAATGGTCTTACATACCAGTTGGTGGATCGTTGCCAAATACAGAGCAGAAAACACTTGCGTTTGGCGCTGCTGCTAGCATGGTTCATCCAGCTACAGGTTATTCAGTTGTCAGATCACTGTCCGAGGCGCCAAAATGCGCCTCAGTACTTGCTAATATTTTAAGACAAAATCATGTCAAGAACATGCTTACTAGTTCAAGTACCACAAGTATCTCAACTCAAGCTTGGAACACCCTTTGGCCACAAGAACGAAAAAGGCAAAGATCGTTTTTCCTATTTGGCTTGGCACTCATATTGCAGTTGGATATTGAGGGGATTAGGTCATTTTTCCGCGCATTCTTCCTTGTGCCAAAATGGATCATTCAAAACACAAAGGAAGATGCGAGCAAGTCTGCCAATAATGTGCTAAAGAAGACAAGAAAGAAAGGAAGAATGCAAAGATCTGTCAATAGAAACAAGAAAGGAAAGTCCAAGAATAAAGAGATTTGTAAAAGATACGACAACCCAGATGACGGAGTATAA

>lcl|Nsyl0187370.1 expressed protein （*Nsyε-LCY*）, similar to Lycopene epsilon cyclase, chloroplastic OS=Solanum lycopersicum GN=CRTL-E-1 PE=2 SV=1

ATGGAGTGTATTGGAGCTCGAAATTTTGCTACAATGGCGGTTTTTACGTGTCCGAGATTCAAATCATTAGGAAGAAAGAGAATTATGCCAAGAAAAAAGCAACCATTTTGGCCTATACATATGAAAGTGAAGTGTAGTGGAAGTGATAGTTGTGTAGTGGTTAAAGAAGATTTTGCTGATGAAGAAGATTACATAAAAGCTGGTGGTTCAGAACTTGTTTTTGTTCAAATGCAGCAGAATAAAGACATGGATCTACAGTCTAAGCTTTCTGATAAGTTGCGACAAATATCATCAGCTGGACAAACTATACTGGATTTAGTGGTCATTGGCTGTGGTCCTGCTGGTCTTGCTCTTGCTGCGGAGTCTGCTAAACTCGGGTTGAACGTGGGGCTCGTTGGTCCTGATCTTCCTTTCACAAATAACTATGGTGTCTGGGAAGACGAGTTCAAAGATCTTGGGCTTCAAGCATGCATTGAACATGTTTGGAGTGATACCATTGTATATCTTGATGATGCCGATCCAATTCTTATTGGACGTGCTTATGGAAGAGTTAGTCGCCATTTACTGCATGAGGAGTTACTCAAAAGGTGTGTGGAGGCAGGTGTTTTATATCTTAACTCGAAAGTGGATAGGATCGTTGAGTCCACAAGTGGCCACAGTCTTGTAGAGTGCGAGGGCGACATTGTCATTCCTTGCAGGTTTGTCACTGTTGCATCTGGAGCCGCCTCAGGGAAATTCTTGCAGTATGAGTTGGGAGGTCCTCGGGTTTCTGTTCAAACAGCTTATGGAGTGGAAGTTGAGGAAACTTGTTTGGCTTCAAAAGATGCAATGCCATTTGATCTATTAAAGAAAAAACTGATGTTACGATTGAACACATTGGGCATAAAAATTAAAAAAATCTACGAGGAGGAATGGTCTTACATACCAGTTGGTGGATCGTTGCCAAATACAGAGCAGAAAACACTTGCGTTTGGCGCTGCTGCTAGCATGGTTCATCCAGCTACAGGTTATTCAGTTGTCAGATCACTGTCCGAGGCGCCAAAATGCGCCTCCGTACTTGCAAATATTTTAAGACAAAATCATGTCAAGAACATGATTACCAGTTCAAGTGCCACAAGTATCTCAACTCAAGCTTGGAACACCCTTTGGCCACAAGAACGAAAAAGGCAACGATCGTTTTTCCTATTTGGATTGGCACTCATATTGCAGCTGGATATTGAGGGGATTAGGTCATTTTTCCGCGCATTCTTCCGTGTACCAAAATGGATGTGGCAAGGTTTTCTTGGCTCTAGTCTTTCATCAGCAGACCTCATGTTATTTGCCTTCTACATGTTTATTATTGCACCAAATGACATGAGAAAAGGCCTAATCAGACATCTGTTATCTGATCCAACTGGTGCAACTATGATAAGAACTTATCTTACATTTTAG

>Solyc12g008980.1.1_CDS__Solanum_lycopersicum_Sly （*Slyε-LCY*）

ATGGAGTGTGTTGGAGCTCAAAATGTTGGAGCAATGGCAGTTTTTACGCGTCCGAGATTGAAACCGTTGGTCGGGAGGAGAGTTATGCCAAGAAAAAAGCAATCTTTTTGGCGTATGAGCAGTATGAAAGTAAAATGTAATAGCAGTAGTGGTAGTGACAGTTGTGTAGTTGATAAAGAAGATTTTGCTGATGAAGAAGATTATATAAAAGCCGGTGGTTCGCAACTTGTATTTGTTCAAATGCAGCAGAAAAAAGATATGGATCAGCAGTCTAAGCTTTCTGATGAGTTACGACAAATATCTGCTGGACAAACCGTACTGGATTTAGTGGTAATCGGCTGTGGTCCTGCTGGTCTTGCTCTTGCCGCGGAGTCAGCTAAATTGGGGTTGAACGTGGGGCTCGTTGGGCCTGATCTTCCTTTCACAAACAACTATGGTGTATGGGAGGACGAGTTCAAAGATCTTGGTCTTCAAGCCTGCATTGAACATGTTTGGCGGGATACCATTGTATATCTTGATGATGATGAACCTATTCTTATTGGCCGTGCCTATGGAAGAGTTAGTCGCCATTTTCTGCACGAGGAGTTACTCAAAAGGTGTGTGGAGGCAGGTGTTTTGTATCTAAACTCGAAAGTGGATAGGATTGTTGAGGCCACAAATGGCCAGAGTCTTGTAGAGTGCGAGGGTGATGTTGTGATTCCCTGCAGGTTTGTGACTGTTGCATCGGGGGCAGCCTCGGGGAAATTCTTGCAGTATGAGTTGGGAGGTCCTAGAGTTTCTGTTCAAACAGCTTATGGAGTGGAAGTTGAGGTTGATAACAATCCATTTGACCCGAGCCTGATGGTTTTCATGGATTATAGAGATTATGTCAGACACGACGCTCAATCTTTAGAAGCTAAATATCCAACATTTCTTTATGCCATGCCCATGTCTCCAACACGAGTCTTTTTCGAGGAAACTTGTTTGGCTTCAAAAGATGCAATGCCATTCGATCTGTTAAAGAAAAAACTGATGCTACGATTGAACACCCTTGGTGTAAGAATTAAAGAAATTTACGAGGAGGAATGGTCTTACATACCGGTTGGTGGATCTTTGCCAAATACAGAACAAAAAACACTTGCATTTGGTGCTGCTGCTAGCATGGTTCATCCAGCCACAGGTTATTCAGTCGTCAGATCACTTTCTGAAGCTCCAAAATGCGCCTCTGTACTTGCAAATATATTACGACAACATTATAGCAAGAACATGCTTACCAGTTCAAGTATCCCGAGTATATCAACTCAAGCTTGGAACACTCTTTGGCCACAAGAACGAAAACGACAAAGATCGTTTTTCCTATTTGGACTGGCTCTGATATTGCAGCTGGATATTGAGGGGATAAGGTCATTTTTCCGCGCATTCTTCCGTGTGCCAAAATGGATGTGGCAGGGATTTCTTGGTTCAAGTCTTTCTTCAGCAGACCTCATGTTATTTGCCTTCTACATGTTTATTATTGCACCAAATGACATGAGAAAAGGCTTGATCAGACATCTTTTATCTGATCCTACTGGTGCAACATTGATAAGAACTTATCTTACATTTTAG

>PGSC0003DMT400000880-CDS （*Stuε-LCY*）

ATGGAGTGTGTTGGAGCTCAAAATGTTGGAGCAATGGCGGTTTTTACGCGTCCGAGATTGAAACCATTAGTTGGGAGGAGAATTATGCCAAGAAAAAAACAATCTTTTTGGCCTATGAGTAGTATGAAAGTGAAATGTAATAGTAGTAGTGGAAGTGAGAGTTGTGTAGTTGATAAAGAAGATTTTGCTGATGAAGAAGATTATATAAAAGCCGGTGGTTCGCAACTTGTTTTTGTTCAAATGCAGCAGAAAAAAGATATGGATCAGCAGTCTAAGCTTTCTGATGAGTTGCAACAAATATCAGCTGGACAAACTGTACTGGATTTAGTGGTTATTGGCTGTGGTCCTGCTGGTCTTGCTCTTGCCGCGGAGTCTGCTAAATTGGGGTTGAACGTGGGGCTCGTTGGGCCTGATCTTCCTTTCACAAATAACTATGGCGTATGGGAGGATGAGTTCAAAGATCTTGGTCTTCAAGCCTGCATTGAACATGTTTGGCGGGATACCATTGTATATCTTGATGATGATGATCCTATTCTTATTGGCCGTGCCTATGGAAGAGTTAGTCGCCATTTACTGCATGAGGAGTTACTCAAAAGGTGTGTGGAGGCAGGTGTTTTGTATCTAAACTCGAAAGTGGATAGGATTGTTGAGGCCACAAATGGCCACAGTCTTGTAGAGTGCGAGGGTGATGTTGTGATTCCCTGCAGGTTTGTGACTGTTGCATCGGGAGCAGCCTCGGGGAAATTCTTGCAGTATGAGTTGGGAGGTCCTAGAGTTTCTGTTCAAACAGCTTATGGAGTGGAAGTTGAGGTCGATAACAATCCATTTGACCCGAGCCTGATGGTTTTCATGGATTATAGAGACTATGTCAGACACGACGCTCAATCTTTAGAAGCTAAATATCCAACATTTCTCTATGCCATGCCCATGTCTCCAACACGAGTCTTTTTCGAGGAAACTTGTTTGGCTTCAAAAGATGCAATGCCATTCGATCTGTTAAAGAAAAAACTGATGTTACGATTGAACACCCTCGGTGTAAGAATTAAAGAAATTTACGAGGAGGAATGGTCTTACATACCAGTTGGAGGATCTTTGCCAAATACAGAACAAAAAACACTTGCATTTGGTGCTGCTGCTAGCATGGTTCATCCAGCCACAGGTTATTCAGTCGTCAGATCACTGTCTGAAGCTCCAAAATGCGCCTTCGTGCTTGCAAATATATTACGACAAAATCATAGCAAGAATATGCTTACTAGTTCAAGTACCCCGAGTATTTCAACTCAAGCTTGGAACACTCTTTGGCCACAAGAACGAAAACGACAAAGATCGTTTTTCCTATTTGGACTGGCTCTGATATTGCAGCTGGATATTGAGGGGATAAGGTCATTTTTCCGCGCGTTCTTCCGTGTGCCAAAATGGATGTGGCAGGGATTTCTTGGTTCAAGTCTTTCTTCAGCAGACCTCATGTTATTTGCCTTCTACATGTTTATTATTGCACCAAATGACATGAGAAGAGGCTTAATCAGACATCTTTTATCTGATCCTACTGGTGCAACATTGATAAGAACTTATCTTACTTTTTAG
