# Supplementary material for: Functional characterization and comparison of lycopene epsilon-cyclase genes in Nicotiana tabacum
Source: BMC Plant Biol. 2022 May 21;22:252. doi: 10.1186/s12870-022-03634-5 (PMC9123772; doi:10.1186/s12870-022-03634-5)
Supplement: Supplementary file 2 — Additional file 2: Figure S1. Sequences and phylogenetic analysis of thelycopene epsilon-cyclase genes in Nicotianatobacum. a Phylogenetic tree andconserved domain analysis of amino acid sequences from Solanaceae species. N. tabacum (Ntε-LCY1), N. tabacum (Ntε-LCY2), Nicotianasylvestris (Nsyε-LCY), Nicotiana tomentosiformis (Ntomε-LCY), Solanum tuberosum (Stuε-LCY),and Solanum lycopersicum (Slyε-LCY) sequences are shown. b Spatial structures, ligand binding sites, andenzyme active sites of Ntε-LCY2 (497 amino acids) and Ntε-LCY1 (475 amino acids) from N.tobacumpredicted with the I-TASSER program. Figure S2. Screening gelelectrophoresis of homozygous mutants of Ntε-LCY2and Ntε-LCY1. a ntε-lcy2-1 homozygousmutant line with two rounds of PCR verification. b ntε-lcy2-2 homozygousmutant line with two rounds of PCR verification. c ntε-lcy1-1 homozygousmutant line with two rounds of PCR verification. d ntε-lcy1-2 homozygousmutant line with two rounds of PCR verification. M, DL2000 DNA marker. FigureS3. Sequencing verification results of bacterial liquidcorresponding to two rounds of PCR reactions for Ntε-LCY2 and Ntε-LCY1homozygous mutant lines. a Partialpeak diagram of WT and two homozygous mutant linesof Ntε-LCY2 (ntε-lcy2-1, ntε-lcy2-2) b Partial peak diagram of WT and twohomozygous mutant lines of Ntε-LCY1 (ntε-lcy1-1, ntε-lcy1-2). Table S1. cis-acting elementsin Ntε-LCY gene promoters (3000 bpupstream of the start codon) identified using PlantCARE online software.Important motifs and their functions are shown to highlight similarities anddifferences among the Ntε-LCYhomologs. Table S2. The primers and targetsequences used to construct the CRISPR/Cas9 vectors for Ntε-LCY2 and Ntε-LCY1 and identify the resulting mutants. Table S3. The primers used for quantitative real-time PCR(qRT-PCR) analysis. [file 12870_2022_3634_MOESM2_ESM.docx]

**
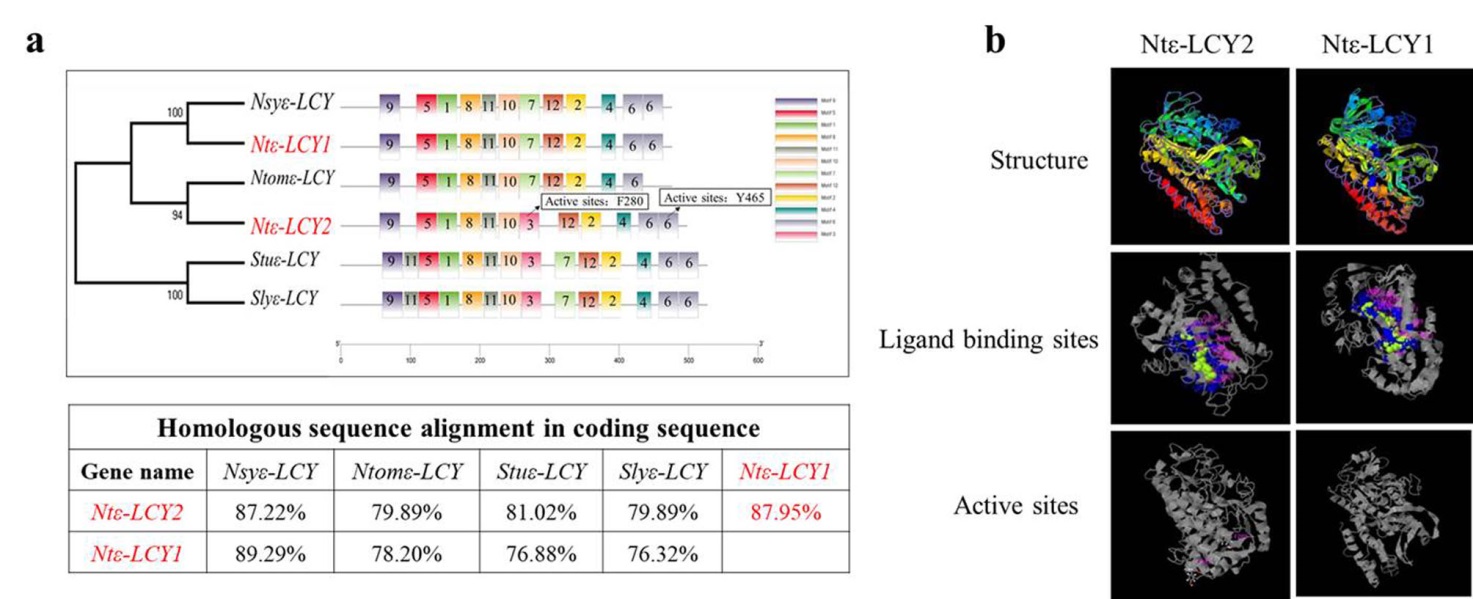
**

**Fig. S1** Sequences and phylogenetic analysis of the lycopene epsilon-cyclase genes in *Nicotiana tobacum*. **a** Phylogenetic tree and conserved domain analysis of amino acid sequences from Solanaceae species. *N. tabacum* (*Ntε-LCY1*), *N. tabacum* (*Ntε-LCY2*), *Nicotiana sylvestris* (*Nsyε-LCY*), *Nicotiana tomentosiformis* (*Ntomε-LCY*), *Solanum tuberosum* (*Stuε-LCY*), *and Solanum lycopersicum* (*Slyε-LCY*) sequences are shown. **b** Spatial structures, ligand binding sites, and enzyme active sites of Ntε-LCY2 (497 amino acids) and Ntε-LCY1 (475 amino acids) from *N. tobacum* predicted with the I-TASSER program.

**a**


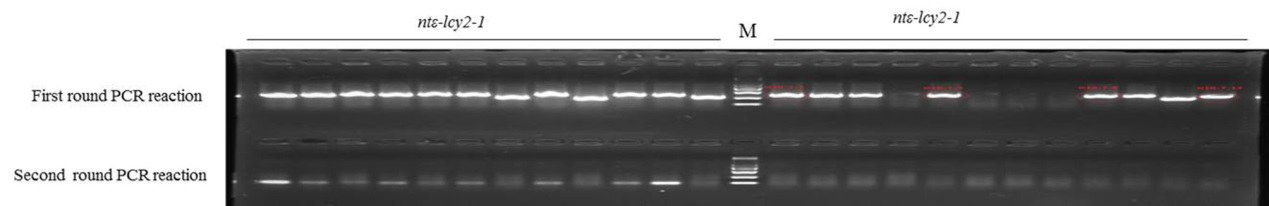


b


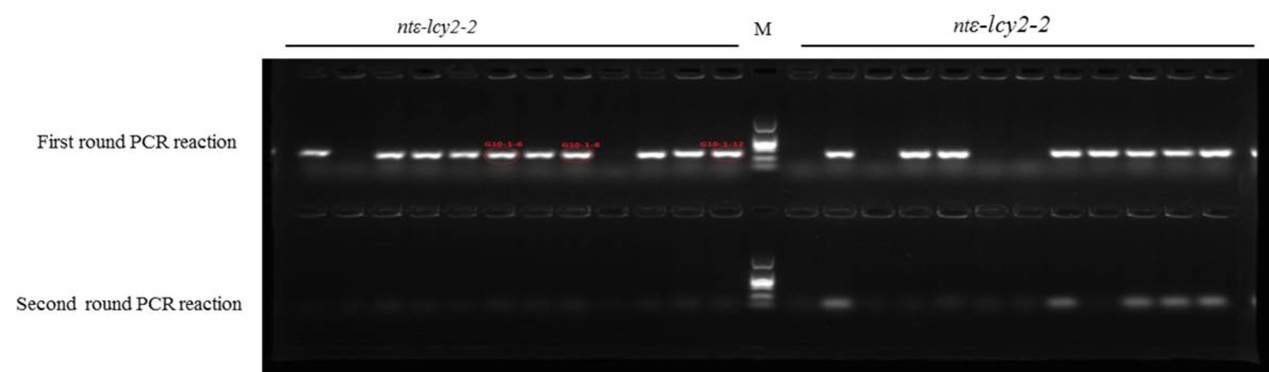


**c**


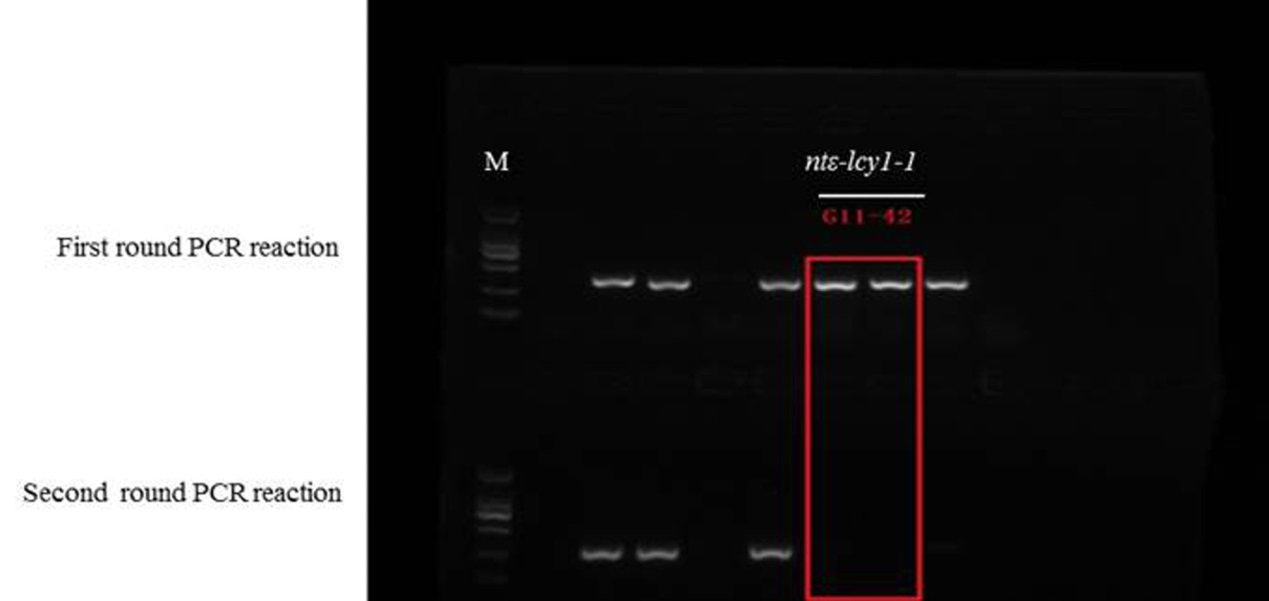


**d**


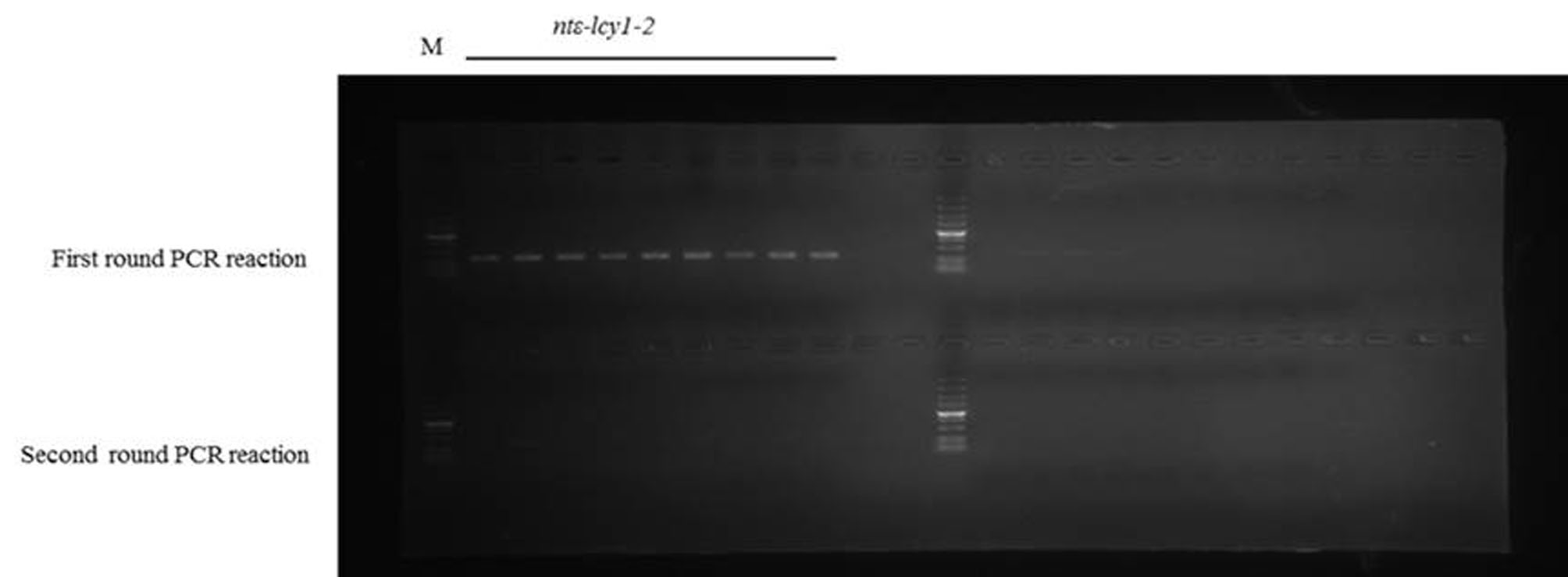


**Fig. S2** Screening gel electrophoresis of homozygous mutants of *Ntε-LCY2* and *Ntε-LCY1*. **a** *ntε-lcy2-1* homozygous mutant line with two rounds of PCR verification. **b** *ntε-lcy2-2* homozygous mutant line with two rounds of PCR verification. **c** *ntε-lcy1-1* homozygous mutant line with two rounds of PCR verification. **d** *ntε-lcy1-2* homozygous mutant line with two rounds of PCR verification. M, DL2000 DNA marker.

**
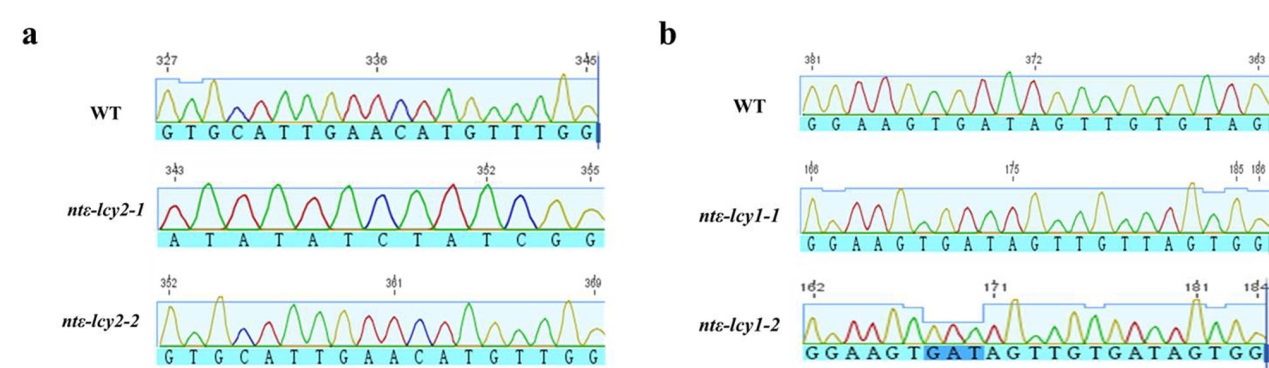
**

**Fig. S3** Sequencing verification results of bacterial liquid corresponding to two rounds of PCR reactions for *Ntε-LCY2* and *Ntε-LCY1* homozygous mutant lines. **a** Partial peak diagram of WT and two homozygous mutant lines of *Ntε-LCY2* (*ntε-lcy2-1*, *ntε-lcy2-2*) **b** Partial peak diagram of WT and two homozygous mutant lines of *Ntε-LCY1* (*ntε-lcy1-1*, *ntε-lcy1-2*).

| Gene | Motif | Function |
| --- | --- | --- |
|  | ARE | anaerobic induction |
|  | MBS | drought inducibility |
|  | ABRE | abscisic acid responsiveness |
|  | AE-box | light responsiveness |
|  | GATA-motif | light responsiveness |
| *Ntε-LCYI & Ntε-LCY2* | G-box | light responsiveness |
|  | Box 4 | light responsiveness |
|  | ATCT-motif | light responsiveness |
|  | GT1-motif | light responsiveness |
|  | TGACG-motif | MeJA responsiveness |
|  | CAT-box | meristem expression |
|  | RY-element | seed-specific regulation |
|  | TGA-element | auxin-responsive element |
|  | circadian | circadian control |
|  | CGTCA-motif | MeJA responsiveness |
| *Ntε-LCY2* | GCN4_motif | endosperm expression |
|  | GA-motif | light responsiveness |
|  | TCT-motif | light responsiveness |
|  | chs-CMA1a | light responsiveness |
|  | chs-CMA2a | light responsiveness |
|  | TC-rich repeats | defense and stress responsiveness |
|  | HD-Zip 1 | differentiation of the palisade mesophyll cells |
| *Ntε-LCY1* | AT1-motif | light responsiveness |
|  | ACE | light responsiveness |
|  | G-Box | light responsiveness |
|  | LTR | low-temperature responsiveness |

**Table S1.** *cis*-acting elements in *Ntε-LCY* gene promoters (3000 bp upstream of the start codon) identified using PlantCARE online software. Important motifs and their functions are shown to highlight similarities and differences among the *Ntε-LCY* homologs.

| **Primer name** | **Primer sequence (5' to 3')** |
| --- | --- |
| Kan-F | CGGCTATGACTGGGCACAACAGACAAT |
| Kan-R | CTCGGCAGGAGCAAGGTGAGATGAC |
| *Ntε-LCY1*-Target | GGAAGTGATAGTTGTGTAG |
| *Ntε-LCY1*-F | TTCAAATCATTAGGAAGAAAGAG |
| *Ntε-LCY1*-R | ACATACCAAACAGTCAATAAGAA |
| *Ntε-LCY2*-Target | GTGCATTGAACATGTTTGG |
| *Ntε-LCY2*-F | AACAACAACATCGTGTAATCTC |
| *Ntε-LCY2*-R | AAATGGCGACTAACTCTTCC |

**Table S2**. The primers and target sequences used to construct the CRISPR/Cas9 vectors for *Ntε-LCY2* and *Ntε-LCY1* and identify the resulting mutants.

| **Primer name** | **Primer sequence (5' to 3')** |
| --- | --- |
| *PSY*-Q-F | TGTTGGAGAAGATGCCAGAAGAG |
| *PSY*-Q-R | ATAAGCAATAGGTAAGGAAATTAGCTTC |
| *PDS*-Q-F | ATAAACCCTGACGAGCTTTC |
| *PDS*-Q-R | AATATGTTCAACAATCGGCAT |
| *ZDS*-Q-F | GAGCATATGCGACAGGATCCCAC |
| *ZDS*-Q-R | TGAAATAGGGGAGCTTGATTTCCGC |
| *CRTISO*-Q-F | CGTGTACACCGAGAATATGATG |
| *CRTISO*-Q-R | GTAGGCGAGAGTCAAGCACTC |
| *β-LCY*-Q-F | GATGACAATACAACTAAAGATCTTGATAG |
| *β-LCY*-Q-R | CATAAGCTACTTGATATCCAGGAT |
| *ε-LCY1*-Q-F | AAAATGCGCCTCCGTACTTG |
| *ε-LCY1*-Q-R | CTTGTGGCCAAAGGGTGTTC |
| *ε-LCY2*-Q-F | TGGTCATAGGCTGTGGTCCT |
| *ε-LCY2*-Q-R | AGAATTGGATCGGCATCGTCA |
| *β-OHase*-Q-F | ATGGCCGCCAGCAGAATTTC |
| *β-OHase*-Q-R | CTCAATTTTCATTTCAATCTCCTCTGTC |
| *VDE*-Q-F | ATGATGCATGGGATGGATATG |
| *VDE*-Q-R | CGTTGGAGCTCTTTAAAACCTTC |
| *ZE*-Q-F | GTGGTGGGATTGGAGGGTTAGTG |
| *ZE*-Q-R | AGGATCTGCTGCAAAGTCATGC |
| *NXS*-Q-F | GCCGGGCTCTATTCGACGTGAT |
| *NXS*-Q-R | ACTGACTCTACCATATGGTCTTCCCAAAT |
| *26S-*RNA-Q-F | GAAGAAGGTCCCAAGGGTTC |
| *26S-*RNA-Q-R | TCTCCCTTTAACACCAACGG |

**Table S3**. The primers used for quantitative real-time PCR (qRT-PCR) analysis.
